# A late Pleistocene gastropod fauna from the northern Caspian Sea with implications for Pontocaspian gastropod taxonomy

**DOI:** 10.3897/zookeys.770.25365

**Published:** 2018-07-04

**Authors:** Thomas A. Neubauer, Sabrina van de Velde, Tamara Yanina, Frank P. Wesselingh

**Affiliations:** 1 Department of Animal Ecology and Systematics, Justus Liebig University, Heinrich-Buff-Ring 26–32 IFZ, 35392 Giessen, Germany; 2 Naturalis Biodiversity Center, P.O. Box 9517, 2300 RA Leiden, The Netherlands; 3 Moscow State University, Faculty of Geography, Leninskie Gory, 1, 119991 Moscow, Russia

**Keywords:** biodiversity, endemism, long-lived lakes, non-marine Gastropoda, Quaternary

## Abstract

The present paper details a very diverse non-marine gastropod fauna retrieved from Caspian Pleistocene deposits along the Volga River north of Astrakhan (Russia). During time of deposition (early Late Pleistocene, late Khazarian regional substage), the area was situated in shallow water of the greatly expanded Caspian Sea. The fauna contains 24 species, of which 16 are endemic to the Pontocaspian region and 15 to the Caspian Sea. The majority of the species (13) belongs to the Pyrgulinae (Hydrobiidae), a group famous for its huge morphological variability in the Pontocaspian region. The phenotypic diversity has led to an inflation of genus and species names in the literature. New concepts are proposed for many of the genera and species found in the present material, with implications for the systematics and taxonomy of the entire Pontocaspian gastropod fauna. *Laevicaspia vinarskii* **sp. n.** is described as a new species. This contribution is considered a first step in revising the Pontocaspian gastropod fauna.

## Introduction

The Caspian Sea is Earth’s largest inland water body. With an area of 378,100 km² it covers about 40% of the world’s continental surface water (Dumont 1998, Lehner and Döll 2004). The endorheic Caspian Basin is situated at the crossroads between Europe and Asia and borders Azerbaijan, Iran, Kazakhstan, Russia, and Turkmenistan. Today, its water balance is strongly controlled by the rivers Volga (Russia) and Ural (Kazakhstan) entering from the north and the Kura River (Azerbaijan) flowing in from the southwest and by evaporation from the sea and the adjacent Kara Bogaz Gol (Dumont 1998). The Caspian Sea is a mesohaline lake with an average salinity of about 12.8‰. Steep salinity gradients exist in the northern Caspian Sea from near freshwater conditions at the Volga River delta in the north to a maximum of 13.8‰ in the southeast (Dumont 1998).

During the Pleistocene, several major transgressive–regressive cycles caused recurrent connections between Black Sea and Caspian basins, which were accompanied by dramatic changes in lake size, salinity and biotic assemblages (e.g., Dumont 1998, Yanko-Hombach et al. 2007, Svitoch 2008, 2012, Shkatova 2010, Forte and Cowgill 2013, Van Baak et al. 2013, Yanina 2013, 2014, Taviani et al. 2014). In spite of the major environmental fluctuations over the geological past, the Caspian Sea Basin hosted a succession of anomalohaline to freshwater lakes since the late Pontian (late Messinian, late Miocene; Popov et al. 2006, Van Baak et al. 2013). The extensive duration facilitated the accumulation of diverse and highly endemic (“Pontocaspian”) biota in this long-lived lake (sensu Gorthner 1994), especially since the Early Pleistocene. As to the recent mollusk fauna, 92 species of gastropods and 35 species of bivalves are listed in latest systematic catalogues (e.g., Kantor and Sysoev 2005, 2006, Kantor et al. 2010, Vinarski and Kantor 2016). As for the gastropods, which are dominated by small-sized Hydrobiidae, 92.4% of them are endemic to the Caspian Sea (Neubauer et al. 2016a). Because of its high diversity, the Caspian Sea has been classified as a major biodiversity hotspot for anomalohaline gastropods (Neubauer et al. 2015a). However, the endemic mollusk fauna is at present severely suffering from the expansion of a number of invasive species (Kosarev and Yablonskaya 1994, Grigorovich et al. 2003, Orlova et al. 2005, Therriault et al. 2004, Riedel et al. 2006, Heiler et al. 2010, Albrecht et al. 2014). Since the early 20^th^ century, human activity has led to a massive increase in the rate of establishment of non-indigenous aquatic species compared to preceding natural colonization (Grigorovich et al. 2003). Additional environmental pressure is exerted on the resident fauna by the increasing concentrations of heavy metals and pesticides (e.g., Agusa et al. 2004, Anan et al. 2005).

In order to predict future biodiversity loss as a response to natural or anthropogenically induced environmental change, it is vital to document and understand the species richness and development of the endemic fauna over longer temporal scales. For this purpose, a sound taxonomic framework is required. The extreme morphological variability of many of the described species complicates taxonomy and, thereby, hampers reliably diversity assessments. Preceding taxonomic studies carried out in the 19^th^ and 20^th^ century have produced a plethora of available species names, partly based on minor morphological deviations. Taxonomic works are hampered by (1) the inadequate nature of descriptions and illustrations, (2) the apparent loss of much of the material, (3) the few and hugely variable morphological characters in some of the groups, and (4) the apparent recent loss of many of the species, which makes combined morphological and molecular approaches impossible. Presently, the statuses of most Caspian endemic gastropods, especially of the numerous representatives of the Pyrgulinae (Hydrobiidae), are poorly resolved.

**Figure 1.**
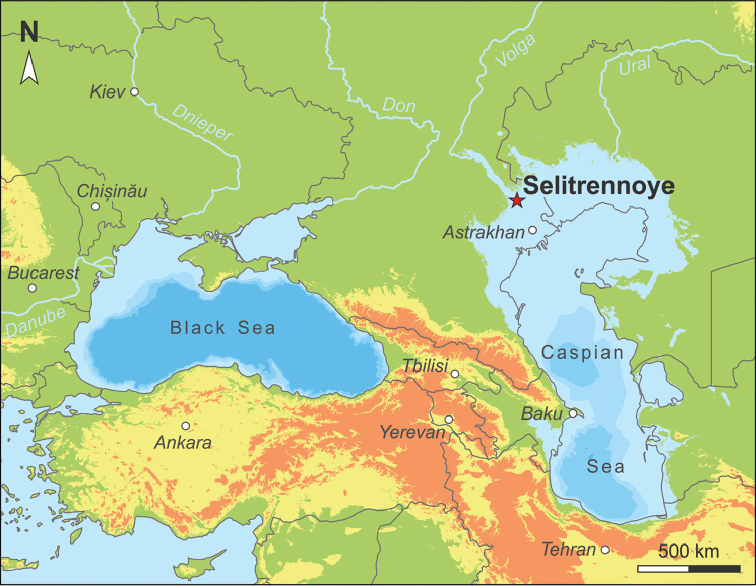
Geographic overview of the Pontocaspian region, with indication of the extent of the late Khazarian (early Late Pleistocene) transgression. The star marks Selitrennoye. Paleo-lake level was modeled in ESRI ArcGIS 10.4 based on Yanina (2014), who suggested an absolute lake level of 10 m b.s.l. at that time. Considering the present Caspian base level of 27 m b.s.l., this estimate corresponds to a lake level rise of 17 m. (Note that the model is restricted to the Pontocaspian catchment area and disregards potential topographic differences.) The bathymetry ranges are based on the GEBCO_2014 model (version 20150318) for present-day (Weatherall et al. 2015); shown isobaths equal to 100, 500 and 1000 m below current lake level.

The present contribution details a diverse gastropod fauna from upper Khazarian (Upper Pleistocene) deposits from the northwestern part of the Caspian Basin, at that time witnessing a major transgressive event (Svitoch 2012; Fig. 1). We provide descriptions, illustrations and comparisons of the so far mostly poorly known species, and suggest nomenclatural and taxonomic rectifications. Since we could examine little of the type material of the discussed species (mostly because the whereabouts are unknown), we limit our conclusions on former concepts and potential synonymies to taxa that have been thoroughly described and/or adequately illustrated (e.g., Kantor and Sysoev 2006). One particular focus of the present work is the revision of genus concepts that have been applied to Pontocaspian Hydrobiidae.

## Materials and methods

**Figure 2.**
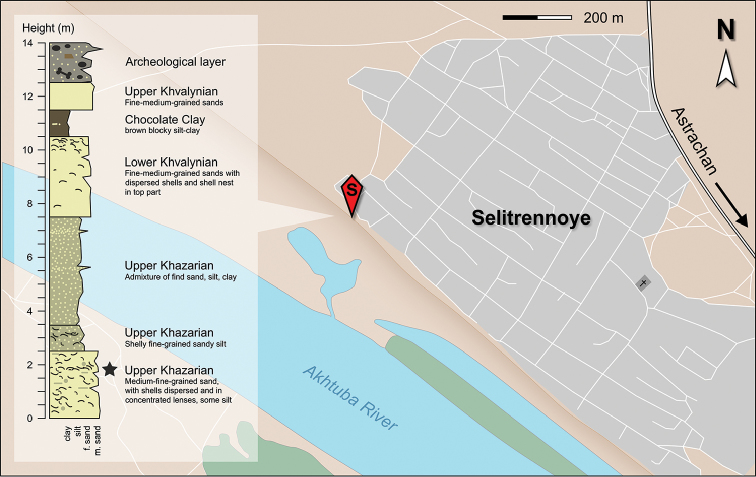
Geographic position and log of the sampled section at Selitrennoye village. The star marks the layer of which the fauna derives. The stratigraphy was established based on the occurrence of Lymnocardiinae bivalves, following the biostratigraphic scheme of Yanina (2013).

The studied mollusk fauna derives from deposits exposed near the small village of Selitrennoye (also as Selitrennoe; *Russ.* Селитренное) along the left bank of the Akhtuba River, a distributary of the Volga River (Russia) (Fig. 1). The locality is situated about 100 km NNW of the city of Astrakhan in the administrative division of the same name (47°10'21.19"N, 47°26'25.41"E, WGS84). The investigated section of 14 m height spans the upper Khazarian to Khvalynian regional substages, which correlates to the early Late Pleistocene (Svitoch 2012, Yanina 2013, 2014). The base of the Quaternary outcrop, which lies 19 m below sea level, is formed by 2.5 m of upper Khazarian sands with common dispersed shells, including shell lenses (Fig. 2). This layer contains the here described gastropod fauna and several species of Lymnocardiinae bivalves. Upsection follows a 1-m-thick interval of horizontally alternating sandy and silty layers containing a diverse assemblage of bivalves of the genera *Monodacna*, *Didacna*, and *Dreissena*. Above it, 4 m of clays containing siltstones and sand layers were deposited. Overlying the interval, 3 m of lower Khvalynian sands are present, containing species of *Didacna* and *Dreissena*, followed by 1 m of brown silty clays (“chocolate clays”). The top of the Pleistocene deposits is marked by 1 m of upper Khvalynian sands and sandy loams barren of fossils, topped by a late Holocene soil complex rich in archeological remains.

Approximately 5 kg of sediment were collected by F.W. in September 2015 and were washed over a 0.5 mm sieve before sorting. All material is stored at the Faculty of Geography of the Moscow State University under collection numbers LV 201501–201530 and 201731–201750 and at the Naturalis Biodiversity Center, Leiden, The Netherlands, under collection numbers RGM 1309784–1309793, 1309797–1309856, 1310190–1310249, and 1310252–1310258.

Macro-photographs of the specimens were taken with a Leica M165 C stereomicroscope with attached DFC420 camera, using the focus stacking function of the Leica Application Suite software v. 4.4.0 at the Naturalis Biodiversity Center, Leiden. SEM images were acquired on a JEOL JSM-6480LV at the same institute. Specimens were coated with a 20 nm thick platinum-palladium alloy in a Quorum Q150T S coater.

**Figure 3.**
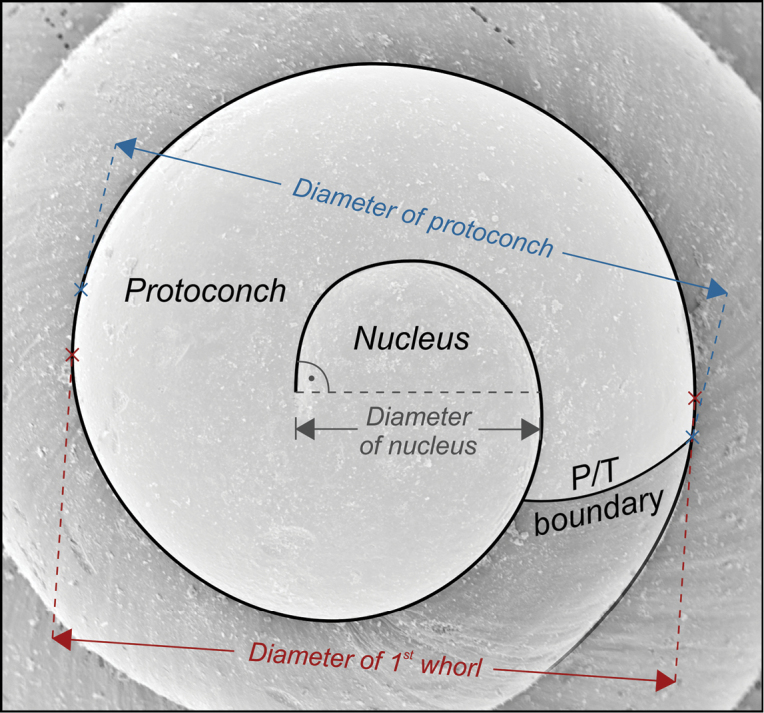
Sketch of the measurements made on the protoconch. The method for counting whorls follows Verduin (1977).

For every species, a number of specimens was measured as representatives of its morphological spectrum. Shell measurements for *Theodoxus* are given as height × largest width (perpendicular to height) × second-largest width (perpendicular to both other axes); for all other species, measurements are given as height × width. Counting of protoconch whorls follows the method used by Verduin (1977) (Fig. 3). Descriptions and information on the whereabouts of type material are only indicated for Pontocaspian species; a brief account on the non-indigenous species detected herein is provided at the end of the Systematic Paleontology section. Synonymy lists comprise original descriptions, records providing illustrations and entries in systematic catalogues referring to Caspian records (e.g., Kantor and Sysoev 2006, Vinarski and Kantor 2016). The systematic classification follows Bouchet et al. (2017) and MolluscaBase (2017).

Abbreviations used are:

**P/T** protoconch/teleoconch;

**MSU** Moscow State University, Moscow, Russia, Faculty of Geography;

**RGM** Naturalis Biodiversity Center, Leiden, The Netherlands, coll. Fossil Mollusca (formerly Rijksmuseum van Geologie en Mineralogie);

**ZIN** Zoological Institute of the Russian Academy of Sciences, St. Petersburg, Russia.

## Systematic paleontology

We tried to locate the depository of type specimens for all identified species, but this was successful only for a part of the fauna. In particular, much of the type material of the species described by Logvinenko and Starobogatov (1969) could not be located, since these authors did not provide information on the depository of the types or the localities they were retrieved at. According to Kantor et al. (2010), the types should be stored in ZIN but few have been found, since large parts of Starobogatov’s collection have not been entirely inventoried as yet.

### Class Gastropoda Cuvier, 1795

#### Subclass Neritimorpha Golikov & Starobogatov, 1975

##### Order Cycloneritimorpha Frýda, 1998

###### Superfamily Neritoidea Rafinesque, 1815

####### Family Neritidae Rafinesque, 1815

######## Subfamily Neritininae Poey, 1852

######### Genus Theodoxus

Taxon classificationAnimaliaCycloneritimorphaNeritidae

Montfort, 1810

########## Type species.

*Theodoxus lutetianus* Montfort, 1810 [currently considered as a synonym of *Theodoxus fluviatilis* (Linnaeus, 1758)]; by original designation. Recent; Europe.

######### Theodoxus pallasi

Taxon classificationAnimaliaCycloneritimorphaNeritidae

Lindholm, 1924

Fig. 4A–F

**Figure 4.**
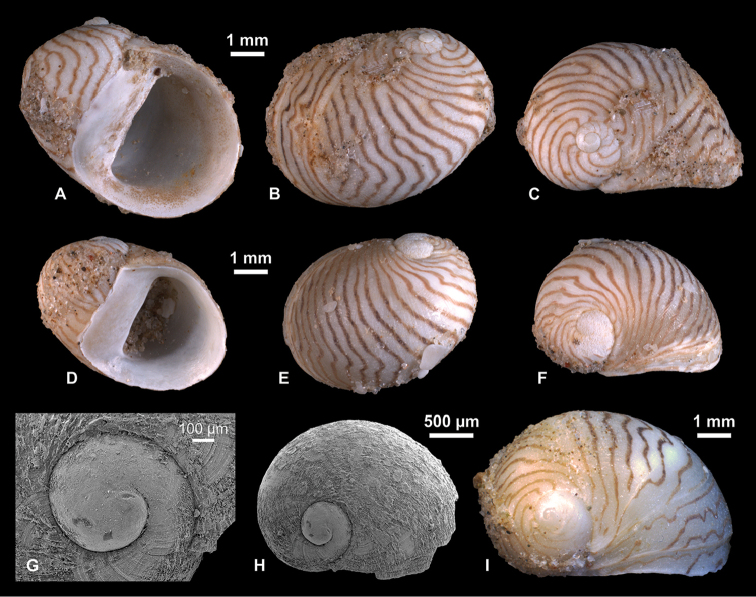
Neritidae . **A–C** *Theodoxus pallasi* Lindholm, 1924, RGM 1310191 **D–F** *T. pallasi*, LV 201510 **G, H** *T. pallasi*, RGM 1309843 **I** *T. pallasi*, RGM 1310192.

1838 Neritina liturata m. Eichwald: 156–157 [non Neritina liturata Schultze, 1826].
1841 Neritina liturata m. – Eichwald: 258–260, pl. 38, figs 18–19 [non Schultze, 1826].
1855 Neritina liturata m. – Eichwald: 307–308 [non Schultze, 1826].
1887 Neritina liturata Eichw. sp. – W. Dybowski: 56–60 [non Schultze, 1826].
1888 [Neritina] liturata Eichw. – W. Dybowski: 79, pl. 2, fig. 10 [non Schultze, 1826].
* 1924 Theodoxus pallasi nom. nov.; Lindholm: 33, 34.
1952 Theodoxus pallasi Lindh. – Zhadin: 208–209, fig. 124.
1969 Theodoxus pallasi Ldh. – Logvinenko & Starobogatov: 343, pl. 5, figs 5–6, textfig. 356.
1994 Theodoxus atrachanicus Starobogatov in Starobogatov et al.: 8–9, fig. 1 (1–2).
1994 *Th.*[*eodoxus*] *pallasi* Ldn. – Starobogatov et al.: 8–9, fig. 1 (3–4).
2006 Theodoxus pallasi Lindholm, 1924. – Kantor & Sysoev: 45, pl. 20, fig. C.
2006 Theodoxus atrachanicus Starobogatov in Starobogatov et al. 1994. – Kantor & Sysoev: 44, pl. 21, fig. C
2009 Theodoxus pallasi Lindholm, 1924. – Filippov & Riedel: 70, 72, 74, 76, figs 4g–i.
2011 Theodoxus astrachanicus Starobogatov in Starobogatov, Filchakov, Antonova et Pirogov, 1994. – Anistratenko et al.: 54–55, fig. 1 (6).
2012 Theodoxus pallasi Lindholm, 1924. – Welter-Schultes: 29, unnumbered textfig.
2016 Theodoxus (Theodoxus) astrachanicus Starobogatov in Starobogatov et al. 1994. – Vinarski & Kantor: 155–156.
2016 Theodoxus (Theodoxus) pallasi (Lindholm, 1924). – Vinarski & Kantor: 156–157.
2017 Theodoxus pallasi Lindholm, 1924. – Anistratenko et al.: 221, figs 4, 7, 10, 11 [cum syn.].

########## Material.

294 specimens (RGM 1309841, RGM 1309843, RGM 1310190–1310193, LV 201510).

########## Type material.

Lectotype: ZIN 54547/63, designated by Starobogatov et al. (1994).

########## Type locality.

“Inter Fucos littoris Derbendensis viva” (living among algae on the shores of Derbent), Dagestan, Russia.

########## Dimensions.

5.95 × 6.62 × 4.81 mm (RGM 1310191, Fig. 4A–C); 4.52 × 5.59 × 4.05 mm (LV 201510, Fig. 4D–F); 6.62 × 7.31 × 5.30 mm (RGM 1310192, Fig. 4I); 6.63 × 7.53 × 4.99 mm (RGM 1310190).

########## Description.

Near globular shell with up to 2.7 whorls. Protoconch consists of about half a whorl; diameter of about 570 µm; nucleus measures ca. 250 µm in diameter; surface mostly corroded; P/T transition indistinct, marked by onset of growth lines. Apex weakly raised. Last whorl passes from upper suture over weakly inclined ramp with shallow concavity into broadly, regularly rounded flank that is near semicircular in profile; relative length of ramp increases with ontogeny. Aperture inclined, regularly semicircular. Callus moderately thickened, glossy, edentate; right margin bulging, symmetrically sinuate, with near straight-sided lower and upper thirds and broad, shallow indentation in central third; left margin extends sinuate over base of penultimate whorl, with small adapical indentation, formed by slightly protruding peristome margin. Peristome sharply edged throughout ontogeny from adapical tip to where it passes into callus margin at base of penultimate whorl. Adapically, peristome margin forms steep crest towards callus, sometimes accompanied by thin, shallow furrow at the transition. Color pattern already starts on early teleoconch as widely spaced, dark yellow to brown curved lines, which pass into slightly irregular zigzag lines with partly dichotomizing branches on last whorl; line width, density, amplitude, color and raggedness varies among specimens and partly within the same individual.

########## Discussion.

The regular, widely spaced zigzag pattern is characteristic of the species. Comparable patterns occur in *T. danubialis* (Pfeiffer, 1828) and occasionally in *T. fluviatilis* (Linnaeus, 1758), but in these species lines are finer and more closely spaced. They furthermore differ in their less elongated shells. Similarly, *T. euxinus* (Clessin, 1886) from the Black Sea is more globular and shows a much denser and finer color pattern (Kantor and Sysoev 2006, Welter-Schultes 2012). *Theodoxus schultzii* (Grimm, 1877) has traditionally been distinguished from *T. pallasi* by its rounder shell and the massively expanded aperture (Zettler 2007). Currently, the whole group is under study using genetic data. Preliminary results suggest that both *T. pallasi* and *T. schultzii* may be grouped with the Armenian species *T. major* Issel, 1865, and possibly a major name change for *T. pallasi* is due (A.F. Sands, pers. commun. 05/2018).

*Theodoxus astrachanicus* Starobogatov in Starobogatov et al. 1994 from the Azov Sea and Volga delta is claimed to differ from *T. pallasi* in size and rate of whorl expansion (Starobogatov et al. 1994). However, both species correspond well in terms of shell shape and, in particular, the typical zigzag pattern (see also Kantor and Sysoev 2006). We therefore agree with Anistratenko et al. (2017) to treat *T. astrachanicus* as a junior synonym of *T. pallasi*.

########## Distribution.

Presently living in the Caspian Sea, the Sea of Azov and the Aral Sea; records from Armenia and the Ural River need confirmation (Anistratenko et al. 2017). In the Pleistocene, the species also dwelled in river deltas entering the Black Sea, where it probably became extinct during the Neoeuxinian/late Pleistocene (Anistratenko et al. 2017).

#### Subclass Caenogastropoda Cox, 1960

##### Order Littorinimorpha Golikov & Starobogatov, 1975

###### Superfamily Truncatelloidea Gray, 1840

####### Family Hydrobiidae Stimpson, 1865

######## Subfamily Caspiinae

Taxon classificationAnimaliaLittorinimorphaHydrobiidae

B. Dybowski, 1913

######### Discussion.

The genus *Caspia* has been widely used for species with small ovoid shells, occasionally with spiral or reticulate teleoconch sculpture. Based on the expression of sculpture, some authors have divided the species among the (sub)genera *Caspia* s.s., with a single spiral line below the suture, and *Clathrocaspia* Lindholm, 1930, exposing a reticulate pattern (e.g., Anistratenko and Prisyazhniuk 1992, Anistratenko 2013, Boeters et al. 2015, Büyükmeriç and Wesselingh 2018). Species lacking teleoconch sculpture were grouped under the new taxon *Ulskia* by Logvinenko and Starobogatov (1969). While those authors considered it a subgenus of *Pyrgula*, W. Dybowski (1887) originally treated its type species (*Caspia ulskii* Clessin & W. Dybowski in W. Dybowski, 1887, see below) as a sculpture-less form of *Caspia*.

*Ulskia ulskii* is available in the present material, and we have investigated the type species of *Clathrocaspia* (*Caspia pallasii* Clessin & W. Dybowski in W. Dybowski, 1887) obtained from Holocene deposits of the northern and southern Caspian Sea. However, the type species of *Caspia*, *Caspia baerii* Clessin & W. Dybowski in W. Dybowski, 1887, is unknown to us. The original description suggests that it is similar to *Ulskia* and *Clathrocaspia* in terms of size and shape, yet to differ in the presence of a single line below to suture, demarcating a narrow subsutural ramp. All three genera are probably closely related, which is also suggested by the similar protoconchs of *Ulskia* and *Clathrocaspia* (pers. obs. T.A.N.). Since *Ulskia* and *Clathrocaspia* can be easily distinguished based on the presence of sculpture, we propose to treat them as distinct genera. The status of *Caspia* remains doubtful until the type species is properly re-investigated.

The *Caspia*–*Clathrocaspia*–*Ulskia* species group can be well delimited from the larger, elongate-conical or -ovoid *Turricaspia* auct. and *Pyrgula* auct. Moreover, unpublished molecular data suggest that the group is unrelated to Pyrgulinae (T. Wilke, pers. comm. 04/2018). We follow Anistratenko (2013) and Bouchet et al. (2017), who listed the Caspiinae as separate subfamily.

######## Genus Ulskia

Taxon classificationAnimaliaLittorinimorphaHydrobiidae

Logvinenko & Starobogatov, 1969

######### Type species.

*Caspia ulskii* Clessin & W. Dybowski in W. Dybowski, 1887; by original designation. Caspian Sea, Recent.

######## Ulskia ulskii

Taxon classificationAnimaliaLittorinimorphaHydrobiidae

(Clessin & W. Dybowski in W. Dybowski, 1887)

Fig. 5A–K

*1887 Caspia Ulskii nob.; W. Dybowski: 38–39.
1888 [Caspia] Ulskii n. sp. – W. Dybowski: 79, pl. 3, fig. 8.
1952 Caspia ulskii W. Dyb., 1888. – Zhadin: 205, fig. 205.
1969 Pyrgula [(Ulskia)] nana Logvinenko & Starobogatov: 379, fig. 367 (12).
1969 Pyrgula [(Ulskia)] schorygini Logv. et Star. sp. n.; Logvinenko & Starobogatov: 379, fig. 367 (11).
1969 Pyrgula [(Ulskia)] ulskii (Cless. et W. Dyb.). – Logvinenko & Starobogatov: 379, figs 367 (10).
2006 Pyrgula nana Logvinenko et Starobogatov, 1968. – Kantor & Sysoev: 101, pl. 47, fig. D.
2006 Pyrgula schorygini Logvinenko et Starobogatov, 1968. – Kantor & Sysoev: 103, pl. 45, fig. E.
2006 Pyrgula ulskii (Clessin et W. Dybowski in W. Dybowski, 1888). – Kantor & Sysoev: 104, pl. 45, fig. F.
2016 Pyrgula nana Logvinenko et Starobogatov, 1968. – Vinarski & Kantor: 240–241.
2016 Pyrgula schorygini Logvinenko et Starobogatov, 1968. – Vinarski & Kantor: 242.
2016 Pyrgula ulskii (Clessin et W. Dybowski in W. Dybowski, 1888). – Vinarski & Kantor: 244.

######### Material.

19 specimens (RGM 1309790, RGM 1309810, RGM 1309856, RGM 1310208, LV 201506).

######### Type material.

“Probable syntype”: ZIN 4608/1. Holotype of *P. schorygini*: ZIN 4357/1. Holotype of *P. nana* not traced.

######### Type locality.

“Kaspi-See” (Caspian Sea, no further details mentioned). Type locality of *P. schorygini*: Caspian Sea; off Apsheron Peninsula, 40°07.5'N, 50°57.5'E, WGS84, 88 m (after Vinarski and Kantor 2016). Type locality of *P. nana*: western part of the Caspian Sea, 70–120 m.

######### Dimensions.

**Figure 5.**
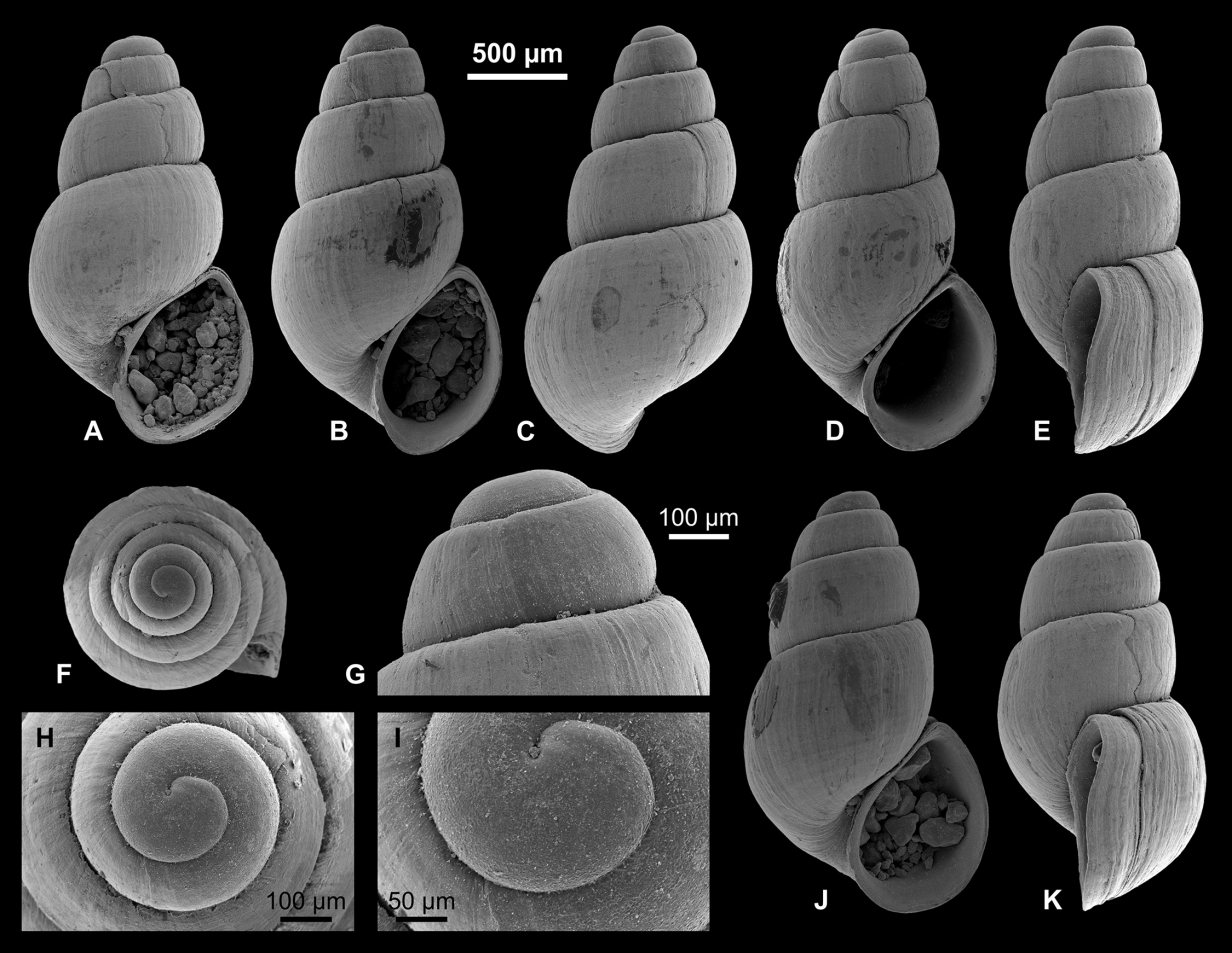
Caspiinae. **A, F, H, I** *Ulskia ulskii* (Clessin & W. Dybowski in W. Dybowski, 1887), RGM 1309810 **B, C, G** *U. ulskii*, LV 201506 **D, E** *U. ulskii*, RGM 1309856 **J, K** *U. ulskii*, RGM 1309790.

2.05 × 1.13 mm (RGM 1309810, Fig. 5A, F, H, I); 2.16 × 1.16 mm (LV 201506, Fig. 5B, C, G); 2.12 × 1.10 mm (RGM 1309856, Fig. 5D, E); 2.12 × 1.23 mm (RGM 1309790, Fig. 5J, K).

######### Description.

Slender ovoid shell with up to 4.7 whorls. Protoconch broad, low dome-shaped, comprising 1.25 whorls that measure 365 µm; nucleus is ca. 105 µm wide; protoconch surface finely but strongly malleate; pattern irregular on initial part and only partly present on nucleus; P/T transition marked by thin axial line and slight step in the upper suture. Teleoconch whorls slightly ton-shaped, weakly convex in abapical half and straight-sided or almost so in adapical half, followed by pronounced convexity at upper suture, producing slightly stepped spire. Last whorl attains ca. 61–66%, descends into steep, straight base. Aperture slender ovoid, slightly inclined, with faint adapical notch at contact to penultimate whorl. Peristome slightly thickened and expanded. In lateral view, outer lip exposes marked adapical indentation and very weak abapical indentation; columellar lip straight. Umbilicus narrow but always open. Growth lines weak but distinctly sigmoidal, with opisthocyrt upper half and prosocline lower half. In addition, faint spiral threads are visible on some shells.

######### Discussion.

*Pyrgula schorygini* Logvinenko & Starobogatov, 1969 and *P. nana* Logvinenko & Starobogatov, 1969, both of which were also originally included in the subgenus Ulskia, closely resemble this species. Logvinenko and Starobogatov (1969) did not discuss similarities or differences among the species involved, but their descriptions suggest they considered minor differences in whorl profile and suture depth sufficient to discriminate species. A similar range of variability is present in our sample as well and might rather reflect intraspecific variation. We thus consider the three species synonymous.

Two more species were attributed to the subgenus Ulskia by Logvinenko and Starobogatov (1969). The shell of *Caspia derzhavini* (Logvinenko & Starobogatov, 1969) is more slender and has more whorls. *Caspia behningi* (Logvinenko & Starobogatov, 1969) differs in its broader and distinctly conical shape.

######### Distribution.

Endemic to the Caspian Sea, reported from water depths between 45 and 120 m (Logvinenko and Starobogatov 1969).

######## ? Subfamily Horatiinae Taylor, 1966

######### Genus Andrusovia

Taxon classificationAnimaliaLittorinimorphaHydrobiidae

Brusina in Westerlund, 1902a

########## Type species.

*Andrusovia dybowskii* Brusina in Westerlund, 1902a; by original designation. Caspian Sea, Recent.

########## Discussion.

The subfamily placement of the genus follows Vinarski and Kantor (2016: 214) and is based on the resemblance with species of the genus *Horatia* Bourguignat, 1887 (see also discussion in Starobogatov 2000). A recent molecular phylogeny including the Hydrobiidae suggests the Horatiinae to be distinct from the Belgrandiinae (Wilke et al. 2013; see also Bank 2017). We follow Starobogatov (2000) and regard *Caspiohoratia* Logvinenko & Starobogatov, 1969 as a junior synonym of *Andrusovia*.

######### Andrusovia brusinai

Taxon classificationAnimaliaLittorinimorphaHydrobiidae

Starobogatov, 2000

Fig. 6F–K, M–N

*2000 Andrusovia brusinai Starobogatov, sp. nov.; Starobogatov: 41, fig. 1C.
2006 Andrusovia brusinai Starobogatov, 2000. – Kantor & Sysoev: 83, pl. 40, fig. C.
2016 Andrusovia brusinai Starobogatov, 2000. – Vinarski & Kantor: 214.

########## Material.

39 specimens (RGM 1309839, RGM 1309840, RGM 1310206, LV 201509).

########## Type material.

Holotype: ZIN (no number).

########## Type locality.

Eastern part of the middle Caspian Sea (42°42.5'N, 51°32.5'E, WGS84), at 80 m.

########## Dimensions.

**Figure 6.**
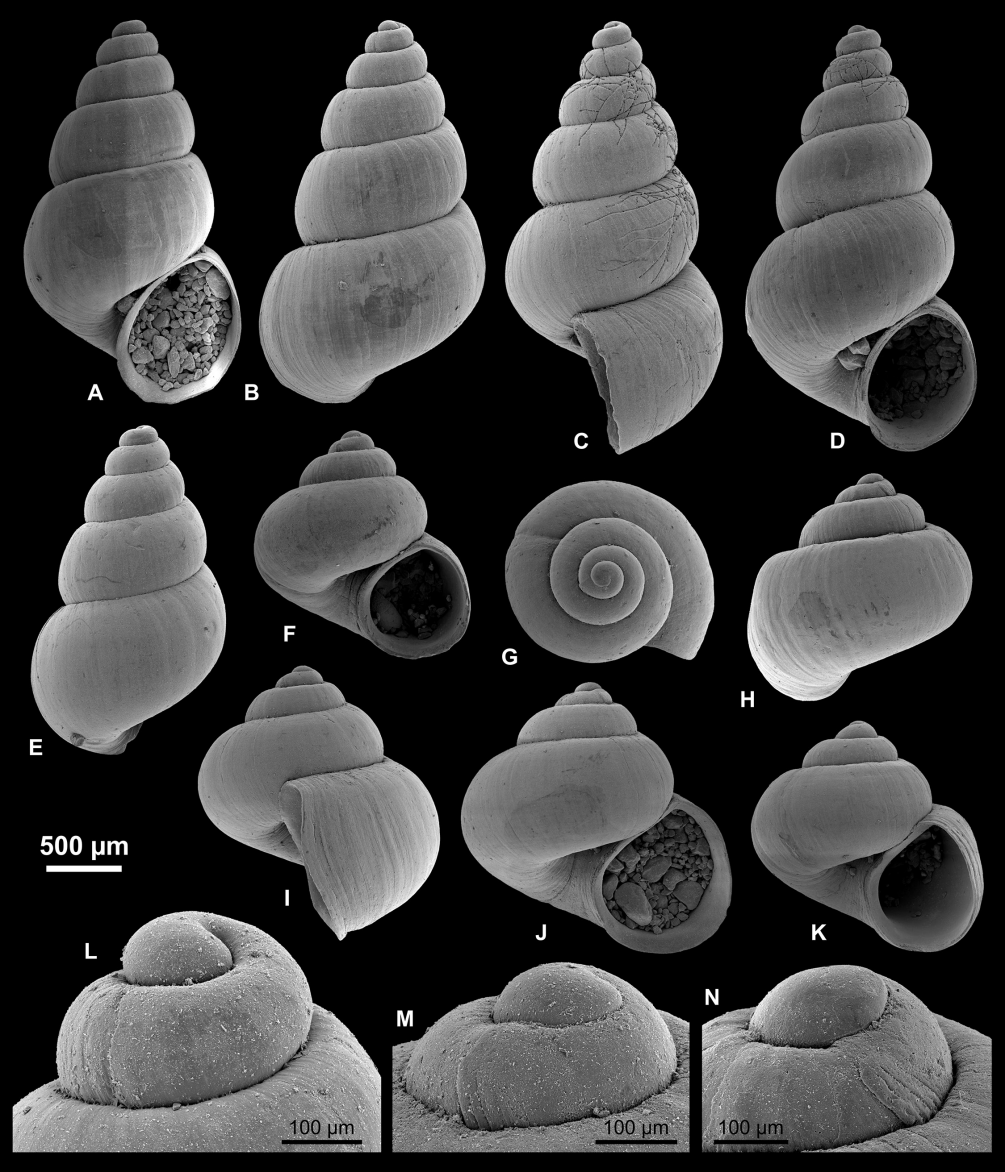
Hydrobiinae and Horatiinae. **A, B, L** Ecrobia cf. grimmi (Clessin in W. Dybowski, 1887), LV 201508 **C, D** E. cf. grimmi, RGM 1309845 **E** E. cf. grimmi, RGM 1309847 **F, G** *Andrusovia brusinai* Starobogatov, 2000, RGM 1309840 **H, K, N** *A. brusinai*, LV 201509 **I, J, M** *A. brusinai*, RGM 1309839.

1.52 × 1.44 mm (RGM 1309840, Fig. 6F, G); 1.54 × 1.55 mm (LV 201509, Fig. 6H, K, N); 1.81 × 1.80 mm (RGM 1309839, Fig. 6I, J, M); 1.71 × 1.52 mm; 1.67 × 1.69 mm; 1.83 × 1.55 mm; 1.64 × 1.51 mm.

########## Description.

Shell broad trochiform, about as high as wide, with up to 4 whorls. Rarely specimens with slightly elevated spire occur. Protoconch high domical, about semicircular in profile; initial part immersed; consists of 1.1 whorls, measures 300 µm in diameter; nucleus about 90 µm wide; protoconch surface finely but strongly malleate near lower suture, rest appears to be irregularly granulate, but that might be due to poor preservation; P/T boundary sharp, marked by massive growth constrictions near lower suture. Teleoconch whorls highly convex, with maximum convexity in adapical half, producing slightly stepped spire. Last whorl attains 74–81% of shell height. Aperture broadly drop-shaped, slightly inclined, with faint adapical notch at contact to penultimate whorl. Peristome slightly thickened and expanded at columella and base; sinuate in lateral view, with weakly protruding central part and weak adapical indentation. Umbilicus wide, deep. Fine prosocline growth lines cover shell. On one specimen, traces of spiral threads occur on base.

########## Discussion.

The Caspian congeners *Andrusovia dybowskii* Brusina in Westerlund, 1902a (sensu Starobogatov 2000) and *A. andrusovi* Starobogatov, 2000 differ from the present species in their much lower spires. *Andrusovia marina* (Logvinenko & Starobogatov, 1969) is smaller and has a shorter spire. Starobogatov (2000) based the distinction from *A. brusinai* on minor differences in shell ratios but these are strongly affected by the varying number of whorls and shell size; it might well be that *A. marina* and *A. brusinai* are just different growth stages of the same species. Since we have not seen the type material of Logvinenko and Starobogatov (1969), we tentatively accept the distinction of both taxa by Starobogatov (2000). Further comparison with the Logvinenko and Starobogatov material is essential to assess whether the two names refer indeed to distinct species.

*Andrusovia brusinai* resembles several recent species of *Horatia* Bourguignat, 1887, *Hauffenia* Pollonera, 1898 and *Islamia* Radoman, 1973 in terms of shell shape and protoconch surface. These differ from the present species in the either straight-sided (*Hauffenia*, *Islamia*; Arconada and Ramos 2006, Erőss and Petro 2008) or abapically (instead of adapically) sinuated peristome (*Horatia*; Szarowska 2006, Szarowska and Falniowski 2014). Shells of several species of *Pontohoratia* Vinarski, Palatov & Glöer, 2015 and *Motsametia* Vinarski, Palatov & Glöer, 2015 resemble *Andrusovia* species in terms of size and shape. They all differ in the more regularly shaped protoconchs, which show large nuclei and lack the massive growth constrictions.

########## Distribution.

Endemic to the Caspian Sea, reported from middle and south Caspian Sea at depths between 47 and 311 m (Starobogatov 2000).

######## Subfamily Hydrobiinae Stimpson, 1865

######### Genus Ecrobia

Taxon classificationAnimaliaLittorinimorphaHydrobiidae

Stimpson, 1865

########## Type species.

*Turbo minutus* Totten, 1834; by original description. United States, Recent.

######### Ecrobia cf. grimmi

Taxon classificationAnimaliaLittorinimorphaHydrobiidae

(Clessin in W. Dybowski, 1887)

Fig. 6A–E, L

cf. * 1887 Hydrobia Grimmi Cless.; W. Dybowski: 55–56.
cf. 1888 [Hydrobia] Grimmi Clessin. – W. Dybowski: 79, pl. 3, fig. 2.
cf. 1952 Hydrobia grimmi (Clessin) W. Dyb., 1888. – Zhadin: 225, fig. 147.
cf. 1969 Pyrgohydrobia grimmi (Cless. et W. Dyb.) – Logvinenko & Starobogatov: 249, fig. 358 (11).
cf. 2006 Caspiohydrobia grimmi (Clessin in W. Dybowski, 1888). – Kantor & Sysoev, 91–92, pl. 43, fig. E.
cf. 2009 Caspiohydrobia grimmi (Clessin et Dybowski, 1888). – Filippov & Riedel: 70–72, 74–76, figs 4a–d.
cf. 2016 Caspiohydrobia grimmi (Clessin et W. Dybowski in W. Dybowski, 1888). – Vinarski & Kantor: 229.

########## Material.

345 specimens (RGM 1309845, RGM 1309847, RGM 1310207, LV 201508).

########## Type material.

Not traced.

########## Type locality.

“Kaspi-See” (Caspian Sea, no further details mentioned).

########## Dimensions.

2.56 × 1.45 mm (LV 201508, Fig. 6A, B, L); 2.83 × 1.54 mm (RGM 1309845, Fig. 6C, D); 2.19 × 1.30 mm (RGM 1309847, Fig. 6E); 3.88 × 2.26 mm; 3.48 × 1.97 mm; 3.77 × 1.99 m; 3.50 × 1.89 mm; 3.26 × 1.79 mm; 3.33 × 1.66 mm.

########## Description.

Shell shape highly variable, ranging from broad ovoid to slender conical, comprising up to 6.5 whorls. Protoconch consisting of about one whorl, with nucleus immersed; initial part slightly raised, producing acute apex; surface weakly granular to malleate; P/T transition clear. Protoconch and teleoconch whorls highly convex, sometimes slightly flattened centrally in later whorls; suture deep. Size of last whorl varies between 55–62%, descends into straight-sided base. Aperture regularly ovoid, slightly inclined, touching base of penultimate whorl, leaving wide umbilicus. Peristome simple, sometimes weakly expanded. Surface smooth expect for very fine prosocline growth lines.

########## Discussion.

The shells of *Ecrobia* can only be reliably identified on the species-level using molecular data (Haase et al. 2010). Therefore, we tentatively assign the detected specimens to *Ecrobia grimmi*, which is the only *Ecrobia* species occurring in the Caspian Sea today (Haase et al. 2010).

Most of the species presently assigned to *Caspiohydrobia* Starobogatov, 1970, including its type species, *Pyrgohydrobia eichwaldiana* Golikov & Starobogatov, 1966, range within the morphological variability of this species. Previous examination of both reproductive systems (Sitnikova et al. 1992) and juvenile shells (Filippov and Riedel 2009) did not yield criteria supporting interspecific differentiation. Very likely all of the thirty *Caspiohydrobia* species listed by Kantor and Sysoev (2006) are morphotypes of a single species, probably *E. grimmi*. Given the problems of using shell morphology to identify *Ecrobia*, taxonomic conclusions on the synonymy of the *Caspiohydrobia* species require molecular data.

########## Note on species authority.

W. Dybowski (1887: 7) noted that all diagnoses were drafted by Clessin and himself and most new species were therefore marked with “nob.” (Lat. *nobis*, “us”). However, W. Dybowski obviously made exceptions. In case of the new genus *Clessinia*, he marked the authority with “m.” (Lat. *meus*, “mine”). For *Hydrobia grimmi*, the authority is clearly indicated with “Cless.”, making Clessin the sole author of the species (unlike indicated by several authors).

########## Distribution.

Caspian Sea; Lake Sawa, Iraq (Haase et al. 2010); salt lakes near Chelyabinsk, Russia (Shishkoedova 2010). Subfossil records derive from Holocene deposits of the Aral Sea (Filippov and Riedel 2009).

######### Subfamily Pyrgulinae

Taxon classificationAnimaliaLittorinimorphaHydrobiidae

Brusina, 1882

1882 Pyrgulinae Brusina: 230.
1914 Micromelaniidae B. Dybowski & Grochmalicki: 276.
1915 Turricaspiinae B. Dybowski & Grochmalicki: 103.
2017 Pyrgulinae Brusina, 1882. – Bouchet et al.: 212, 346 [cum syn.].

########## Discussion.

The Caspian Pyrgulinae (sensu lato) encompasses 64 species that are considered accepted in the current literature (Vinarski and Kantor 2016). However, most of them are poorly known, documented by insufficient descriptions and drawings; for many, the type material has not been found (Kantor and Sysoev 2006, Vinarski and Kantor 2016). The extreme morphological variability of several representatives, such as those detected in the material from Selitrennoye, led previous authors to introduce numerous species based on shells with only minor deviations in shape, size or whorl outline. The Caspian Pyrgulinae therefore requires careful revision using molecular and anatomical data as far as available.

In addition to the problems associated with distinguishing species, genus-level classification is poorly resolved as well. Several attempts have been made to categorize this vast variability, and genus concepts have changed tremendously (e.g., B. Dybowski and Grochmalicki 1915, 1917, Zhadin 1952, Logvinenko and Starobogatov 1969, Kantor and Sysoev 2006, Vinarski and Kantor 2016). Twelve genus names have been described for members of the Caspian Pyrgulinae, based on quite different concepts of traits considered diagnostic. Currently, all species are classified within *Caspia* Clessin & W. Dybowski, 1887, *Pyrgula* De Cristofori & Jan, 1832 and *Turricaspia* B. Dybowski & Grochmalicki, 1915 (Kantor and Sysoev 2006, Vinarski and Kantor 2016). This scheme unites quite a variety of different morphologies under the same genus names, while at the same time similar species are assigned to different genera (e.g., Kantor and Sysoev 2006). Unfortunately, hardly any previous study provided explanations for their genus classifications or systematic concepts in general.

A thorough revision of all Caspian Pyrgulinae is beyond the scope of this study, but we discuss and revise the concepts that have been applied to the species studied herein.

Vinarski and Kantor (2016) listed 38 species of the genus *Pyrgula* for the Caspian Sea. The type species of *Pyrgula* De Cristofori & Jan, 1832, *P. annulata* (Linnaeus, 1758), lives in freshwater lakes and springs in Italy and Dalmatia (Welter-Schultes 2012). Shell morphology, anatomy and protoconch characteristics are very similar to Pontocaspian Pyrgulinae, e.g., some species of *Turricaspia* (compare also discussion in Riedel et al. 2001). However, molecular evidence suggests that *Pyrgula annulata* is only distantly related to the Pontocaspian species flock within the Pyrgulinae, with the last common ancestor dating back to the late Miocene (Wilke et al. 2007). Therefore, Pontocaspian species should not be attributed to *Pyrgula*, despite apparent morphological congruence, especially of some of the keeled Pontocaspian Pyrgulinae. A separation on subfamily level as proposed by B. Dybowski and Grochmalicki (1915) is opposed by the latest phylogeny of rissooidean gastropods, which suggests a rather close relationship (Wilke et al. 2013).

*Turricaspia* B. Dybowski & Grochmalicki, 1915 (type species: *Micromelania turricula* B. Dybowski & Grochmalicki, 1915) was introduced for species with turriform, elongate shells with numerous whorls. Presently, the genus includes 22 Caspian species, encompassing elongate and broad, conical and ovoid, and sculptured and smooth species (Kantor and Sysoev 2006, Vinarski and Kantor 2016). Many species assigned to *Pyrgula* by Kantor and Sysoev (2006) and Vinarski and Kantor (2016) actually resemble *Turricaspia turricula* with respect to the turriform, conical shell. This similarity also regards the type species of the genera *Caspiopyrgula* Logvinenko & Starobogatov, 1969 (type species: *Turricaspia nossovi* Kolesnikov, 1947), *Eurycaspia* Logvinenko & Starobogatov, 1969 (*Micromelania pseudodimidiata* B. Dybowski & Grochmalicki, 1917), *Oxypyrgula* Logvinenko & Starobogatov, 1969 (*Pyrgula pseudospica* Logvinenko & Starobogatov, 1969), and *Trachycaspia* B. Dybowski & Grochmalicki, 1917 (*Rissoa dimidiata* Eichwald, 1838). After examination of descriptions and illustrations of the type species (e.g., Kantor and Sysoev 2006), we conclude that these genera should be considered as junior synonyms of *Turricaspia*.

Some of the species classified as *Turricaspia* by Kantor and Sysoev (2006) and Vinarski and Kantor (2016) differ considerably from *Turricaspia* s.s. in shell shape. This contains the type species of the genera *Caspiella* Thiele, 1928 (*Rissoa conus* Eichwald, 1838), *Clessiniola* Lindholm, 1924 (*Paludina variabilis* Eichwald, 1838), and *Laevicaspia* B. Dybowski & Grochmalicki, 1917 (*Rissoa caspia* Eichwald, 1838). In turn, some species presently attributed to the genus *Euxinipyrgula* Sitnikova & Starobogatov, 1999 (type species: *Pyrgula milachevitchi* Golikov & Starobogatov, 1966) closely resemble species of the *Laevicaspia*–*Caspiella* group (compare Anistratenko et al. 2011).

Based on a review of the Pontocaspian species formerly attributed to these genera and illustrated in the literature (Golikov and Starobogatov 1966, Logvinenko and Starobogatov 1969, Alexenko and Starobogatov 1987, Kantor and Sysoev 2006, Anistratenko 2008), we propose to distinguish the genera *Clessiniola* and *Laevicaspia* from *Turricaspia*, and to treat *Caspiella* and *Euxinipyrgula* as junior synonyms of *Laevicaspia*.

*Clessiniola* species can be easily distinguished from species attributed to other genera based on their broad shells with a large body whorl and aperture. The situation for the *Laevicaspia*–*Caspiella*–*Euxinipyrgula* is more difficult. The three type species (see above) share the ovoid shape with cyrtoconoid spire, the high whorl accretion rate, the shape, inclination, lateral sinuation and thickening of the aperture, and the extent and sculpture of the protoconch (e.g., Kantor and Sysoev 2006, Anistratenko 2008, and this study). The only differences are shell size and whorl convexity, which we do not consider sufficient to distinguish genera. The adapical thickening of the aperture resulting from downward growing of the shell in late ontogeny as stated in the diagnosis of the genus *Euxinipyrgula* by Sitnikova and Starobogatov (1999) is also shown for species that have been attributed to *Caspiella* (see below). The features of the soft-part anatomy considered diagnostic by these authors need to be rechecked and compared to live material from the Caspian Sea to reevaluate the position of *Euxinipyrgula*. Sitnikova and Starobogatov (1999) also discussed the similarities between *Caspiella* and *Euxinipyrgula*, concluding that *Caspiella* should perhaps be included in the genus *Euxinipyrgula*, possibly as a separate subgenus (which would be nomenclaturally invalid however).

The ovoid shape, lateral sinuation and thickening of the aperture typical for the *Laevicaspia*–*Caspiella*–*Euxinipyrgula* group are also found among species of the genus *Prososthenia* Neumayr, 1969 from the middle Miocene of the Dinaride Lake System (e.g., Neubauer et al. 2016b). These species, however, differ in the granulate protoconch making up less than one whorl.

Species of *Turricaspia* differ from *Laevicaspia* in the slower, regular whorl accretion, producing a conical spire and a higher number of whorls at the same size. In addition, *Turricaspia* species have usually more fragile shells, thinner peristomes and often more strongly sinuate growth lines.

The genus *Caspia* is listed among Pyrgulinae in latest catalogues (Kantor and Sysoev 2006, Vinarski and Kantor 2016), but it has been shown to be unrelated to that subfamily (Anistratenko 2013, Bouchet et al. 2017; see discussion of the Caspiinae above).

Finally, several Pontocaspian Pyrgulinae have been previously assigned to the genus *Micromelania* Brusina, 1874 (e.g., W. Dybowski 1887, B. Dybowski and Grochmalicki 1917). Its type species, *Micromelania cerithiopsis* Brusina, 1874 (subsequent designation by Dollfus 1912), derives from late Miocene deposits of Lake Pannon. It differs considerably from Pontocaspian Pyrgulinae regarding the presence of 2–4 noded keels and the small size (4.5 × 1.33 mm after Brusina 1874) compared to the rather high number of eight whorls.

######### Genus Clessiniola

Taxon classificationAnimaliaLittorinimorphaHydrobiidae

Lindholm, 1924

1887 Clessinia W. Dybowski: 41 [non Doering, 1875].
1924 Clessiniola Lindholm: 32–33, 34.
1928 Clessinola Strand: 68 [junior objective synonym of Clessiniola].

########## Type species.

*Paludina variabilis* Eichwald, 1838; by typification of replaced name (*Clessinia* W. Dybowski, 1887). Volga delta and Caspian Sea, Quaternary to Recent.

######### Clessiniola variabilis

Taxon classificationAnimaliaLittorinimorphaHydrobiidae

(Eichwald, 1838)

Fig. 7A–I

*1838 Paludina variabilis m.; Eichwald: 151–152.
1841 Paludina variabilis m. – Eichwald: 253–254, pl. 38, figs 6–7.
1853 *Pal.*[*udina*] *variabilis* m. – Eichwald: 285.
1887 Clessinia variabilis Eichw. sp. – W. Dybowski: 41–42.
1888 [Clessinia] variabilis Eichw. sp. – W. Dybowski: 79, pl. 2, fig. 6.
1952 Clessiniola variabilis (Eichwald, 1841). – Zhadin: 255, fig. 199.
1966 *P.*[*yrgula*] (*Clessiniola*) *variabilis*. – Golikov & Starobogatov: 356, fig. 2 (2).
1969 Pyrgula [(Clessiniola)] variabilis (Eichw.) – Logvinenko & Starobogatov: 377, fig. 367 (1).
1987 *T.*[*urricaspia*] *variabilis* (Eichw.). – Alexenko & Starobogatov: 34, fig. 5.
2006 Turricaspia variabilis (Eichwald, 1838). – Kantor & Sysoev: 111, pl. 49, fig. J.
2011 Turricaspia variabilis (Eichwald, 1838). – Anistratenko et al.: 85, fig. 3 (15).
2014 Turricaspia variabilis. – Taviani et al.: 4, fig. 3b.
2016 Turricaspia variabilis (Eichwald, 1838). – Vinarski & Kantor: 251.

########## Material.

4867 specimens (RGM 1309815, RGM 1309826, RGM 1309827, RGM 1309831, RGM 1310243–1310247, LV 201507).

########## Type material.

Not traced.

########## Type locality.

“In Volgae ostio prope Astrachanum, et versus mare Caspium; etiam fossili in calcatio lapide conglutinato recentissimo Dagesthanici littoris” (at the Volga river mouth near Astrakhan, and towards the Caspian Sea; also in recently lithified fossil limestone at the shores of Dagestan).

########## Dimensions.

**Figure 7.**
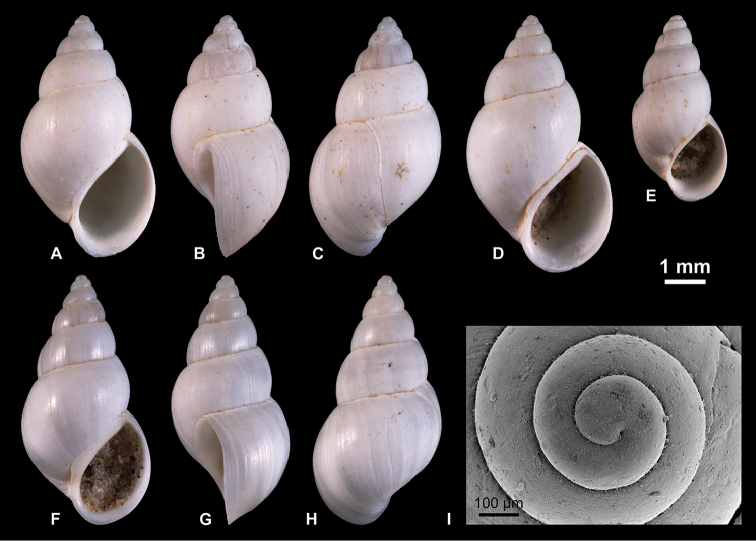
Pyrgulinae. **A–C** *Clessiniola variabilis* (Eichwald, 1838), LV 201507, broad morphotype **D** *C. variabilis*, RGM 1310246, broad morphotype **E** *C. variabilis*, RGM 1310245, slender morphotype **F–H** *C. variabilis*, RGM 1310243, slender morphotype **I** *C. variabilis*, RGM 1309827.

5.91 × 3.31 mm (LV 201507, Fig. 7A–C); 6.31 × 3.59 mm (RGM 1310246, Fig. 7D); 4.60 × 2.35 mm (RGM 1310245, Fig. 7E); 6.08 × 3.18 mm (RGM 1310243, Fig. 7F–H); 6.85 × 3.89 mm (RGM 1310244).

########## Description.

Broadly drop-shaped to rarely conical shell of up to six whorls. Protoconch insufficiently preserved to specify extent and surface sculpture; P/T transition indistinct; first whorl measures ca. 340 µm in diameter. Teleoconch whorls moderately and regularly convex; sometimes, spire is very faintly stepped; suture narrow. In many specimens, shells starts to grow stronger in abapical direction in course of last (two) whorl(s), producing non-parallel suture and relatively higher penultimate whorl. Rarely, forms with comparatively slender shape and regularly increasing whorls (and thus relatively smaller last and penultimate whorls) occur. Both types are linked via intermediates. Aperture regularly ovoid, inclined; inner lip glossy, weakly to sometimes more prominently thickened; strongly adnate, sheet-like expanded over base of penultimate whorl and columella, rarely leaving very narrow umbilicus; broad, shallow spout occurs at transition between columella and base; outer lip mainly thin, sometimes weakly thickened at anterior notch. Growth lines very faint, with prosocline upper third and near orthocline lower two-thirds.

########## Discussion.

This species displays a large morphological variability within our ample material. Shell shape ranges between slender conical to broadly ovoid, sometimes with weakly irregular growth. Likewise, shell size, whorl convexity, and number of whorls vary considerably. Yet, these features intergrade without clear boundary, rendering a distinction of species unreasonable.

The morphological variability is not restricted to our material but a general feature of *Clessiniola*. It was documented by several previous authors, partly for specimens from the same localities (e.g., Eichwald 1838, Issel 1865, W. Dybowski 1887–1888, Golikov and Starobogatov 1966, Logvinenko and Starobogatov 1969, Alexenko and Starobogatov 1987, Anistratenko et al. 2011). The species concepts applied by the different authors, however, varied greatly. The present material includes shells that have been variably attributed to the species *C. variabilis* (Eichwald, 1838), *C. triton* (Eichwald, 1838) and *C. martensii* (Clessin & W. Dybowski in W. Dybowski, 1887). The *triton*-morphotype sensu Eichwald characterizes broad specimens with slightly detached aperture (see also Kantor and Sysoev 2006); these forms are rarely represented in our material. Eichwald (1838, 1841) himself confirmed the rarity of the form, also stating that he did not find a living representative (in contrast to *C. variabilis*). The *martensii*-morphotype was introduced for similarly broad morphologies. (Note that Clessin and W. Dybowski used a different concept of *C. triton*, there having a rather elongate conical shell.)

Because of the fluent morphological transition between forms traditionally referred to as *C. variabilis*, *C. triton* and *C. martensii*, as well as their joint occurrence in several localities in the Pontocaspian region, one might consider all of them synonymous. Personal observations on Holocene material from Dagestan area, however, indicate indeed distinguishable morphotypes without intermediates. Moreover, frequent shell repair found in most of the Selitrennoye specimens additionally complicates an unbiased view on morphological diversity. A more in-depth investigation comparing undamaged material from different sites is thus required.

Given the large variability, the Caspian species *Clessiniola ovum* (Logvinenko & Starobogatov, 1969) and *C. trivialis* (Logvinenko & Starobogatov, 1969), as well as *C. pseudotriton* (Golikov & Starobogatov, 1966) from the Dniester River mouth (compare Kantor and Sysoev 2006), might too be considered as synonyms of *C. variabilis*. However, the original descriptions and drawings provided impede clarification of their statuses.

*Clessinia ahngeri* Westerlund, 1902 is often listed as junior synonym of *C. variabilis*, but without discussion (e.g., Logvinenko and Starobogatov 1969, Vinarski and Kantor 2016). The original description of *C. ahngeri* suggests close similarities indeed between both species claiming, however, that it differs from other congeners in the much larger spire (11 × 5 mm) and the slightly sinuate outer lip. Examination of Westerlund’s (1902b) material is required to ascertain the alleged synonymy.

The record of “*Paludina Eichwaldi* Kryn.” Eichwald (1841) listed in synonymy of *C. variabilis* refers to a nomen nudum mentioned in a species list by Krynicki (1837).

########## Distribution.

Endemic to the Pontocaspian region. Found in the Caspian Sea and the lower courses of rivers and freshwater parts of the Azov and Black seas (Anistratenko et al. 2011, Vinarski and Kantor 2016). Also reported from Neoeuxinian (late Pleistocene) deposits of the Marmara Sea (Taviani et al. 2014).

######### Genus Laevicaspia

Taxon classificationAnimaliaLittorinimorphaHydrobiidae

B. Dybowski & Grochmalicki, 1917

? 1902a Thaumasia Westerlund: 104 [non Perty, 1833; non Albers, 1850].
1917 LaevicaspiaB. Dybowski & Grochmalicki: 5.
1928 Caspiella Thiele: 353, 381.
1999 Euxinipyrgula Sitnikova & Starobogatov: 158, 162.

########## Type species.

*Rissoa caspia* Eichwald, 1838; by subsequent designation by Logvinenko and Starobogatov (1969). Caspian Sea, Pleistocene.

########## Discussion.

Lindholm (1922) studied the type material of *Buliminus goebeli* Westerlund, 1896 from Mangyschlak (Mangystau Peninsula, Kazakhstan) and concluded that is a junior synonym of “*Micromelania*” *caspia* (Eichwald, 1838). Westerlund (1902a), considering *Buliminus goebeli* as a member of terrestrial “Bulimoidea” (= Enidae), introduced the new genus *Thaumasia*, which would take precedence over *Laevicaspia* B. Dybowski & Grochmalicki, 1917. However, *Thaumasia* Westerlund, 1902 is invalid as a junior homonym of *Thaumasia* Perty, 1833 (Arachnida) and *Thaumasia* Albers, 1850 (Gastropoda, Subulinidae) (see also Lindholm 1925).

######### Laevicaspia caspia

Taxon classificationAnimaliaLittorinimorphaHydrobiidae

(Eichwald, 1838)

Fig. 8A–K

**Figure 8.**
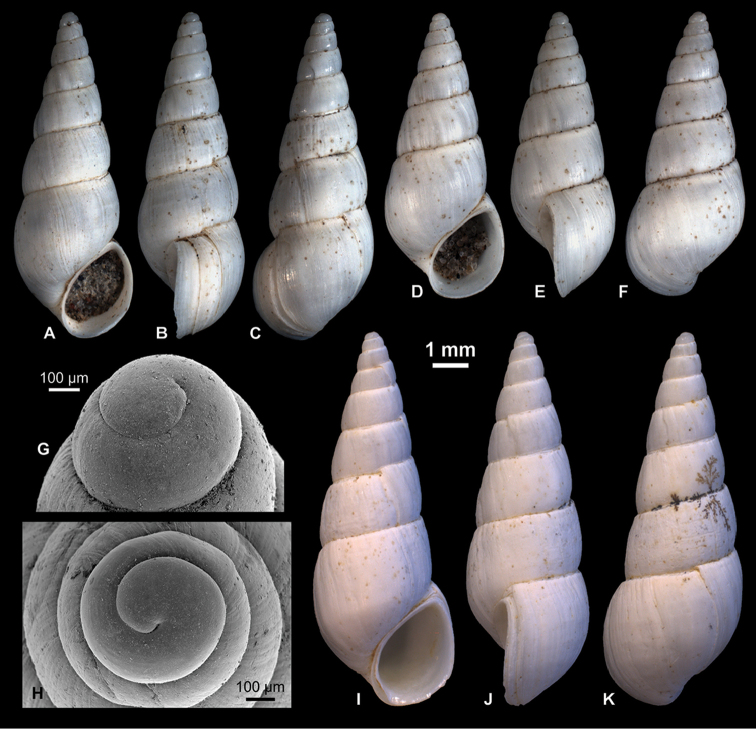
Pyrgulinae. **A–C** *Laevicaspia caspia* (Eichwald, 1838), RGM 1310257 **D–F** *L. caspia*, RGM 1310258 **G** *L. caspia*, RGM 1310197 **H** *L. caspia*, RGM 1310198 **I–K** *L. caspia*, LV 201511.

**Figure 9.**
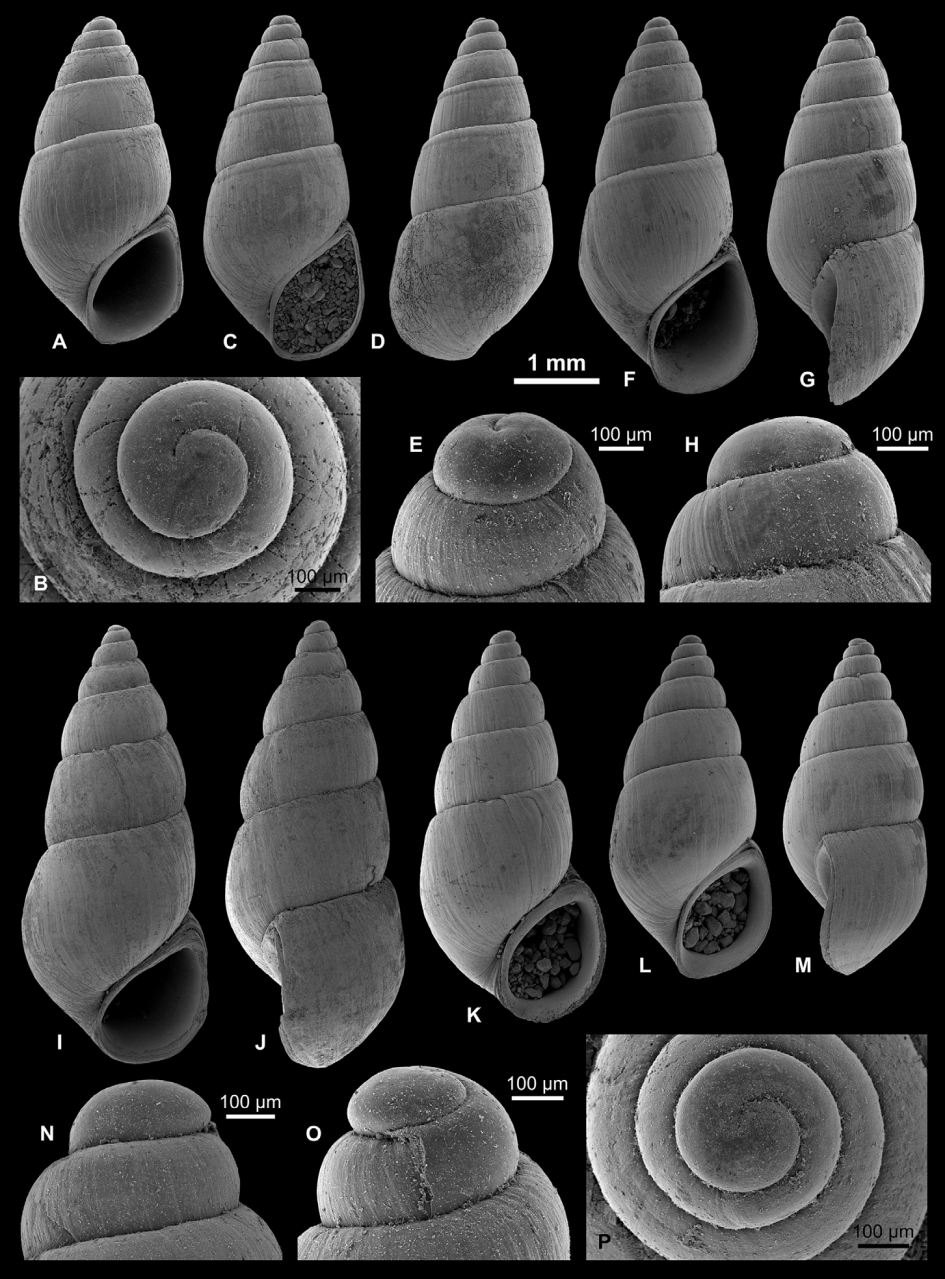
Pyrgulinae. **A, B** *Laevicaspia cincta* (Abich, 1859), LV 201514 **C–E** *L. cincta*, RGM 1309807 **F–H** *L. cincta*, RGM 1309806 **I, J** *L. cincta*, RGM 1309830 **K** *Laevicaspia conus* (Eichwald, 1838), LV 201515 **L–O** *L. conus*, RGM 1309829 **P** *L. conus*, RGM 1309828.

**Figure 10.**
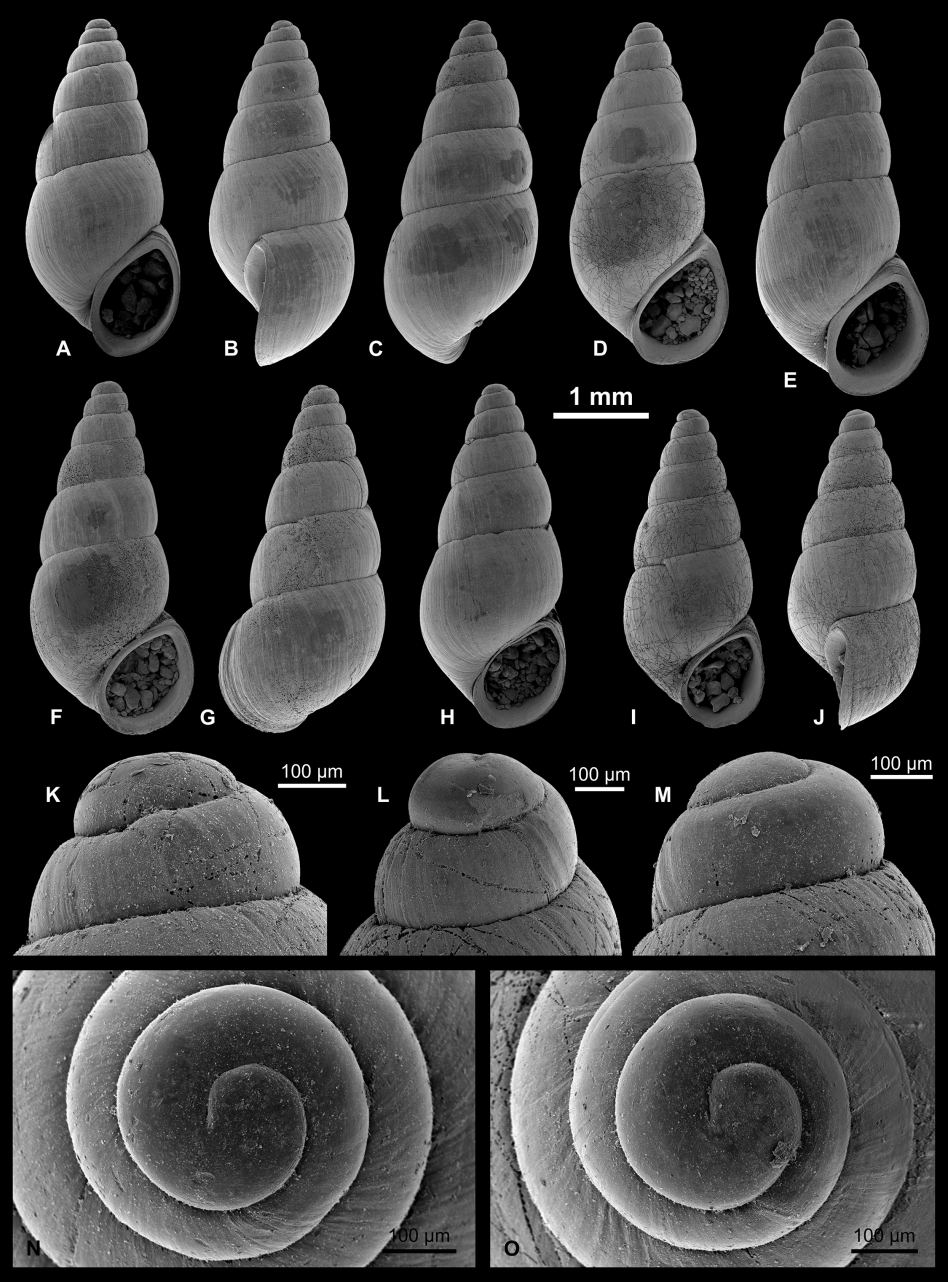
Pyrgulinae. **A–C, K** *Laevicaspia kolesnikoviana* (Logvinenko & Starobogatov in Golikov & Starobogatov, 1966), RGM 1309816 **D** *L. kolesnikoviana*, LV 201516 **E, N** *L. kolesnikoviana*, RGM 1309819 **F, G** *Laevicaspia vinarskii* sp. n., holotype, LV 201517 **H, O** *L. vinarskii* sp. n., RGM paratype, 1309805 **I, J, L, M** *L. vinarskii* sp. n., paratype, RGM 1309821.

**Figure 11.**
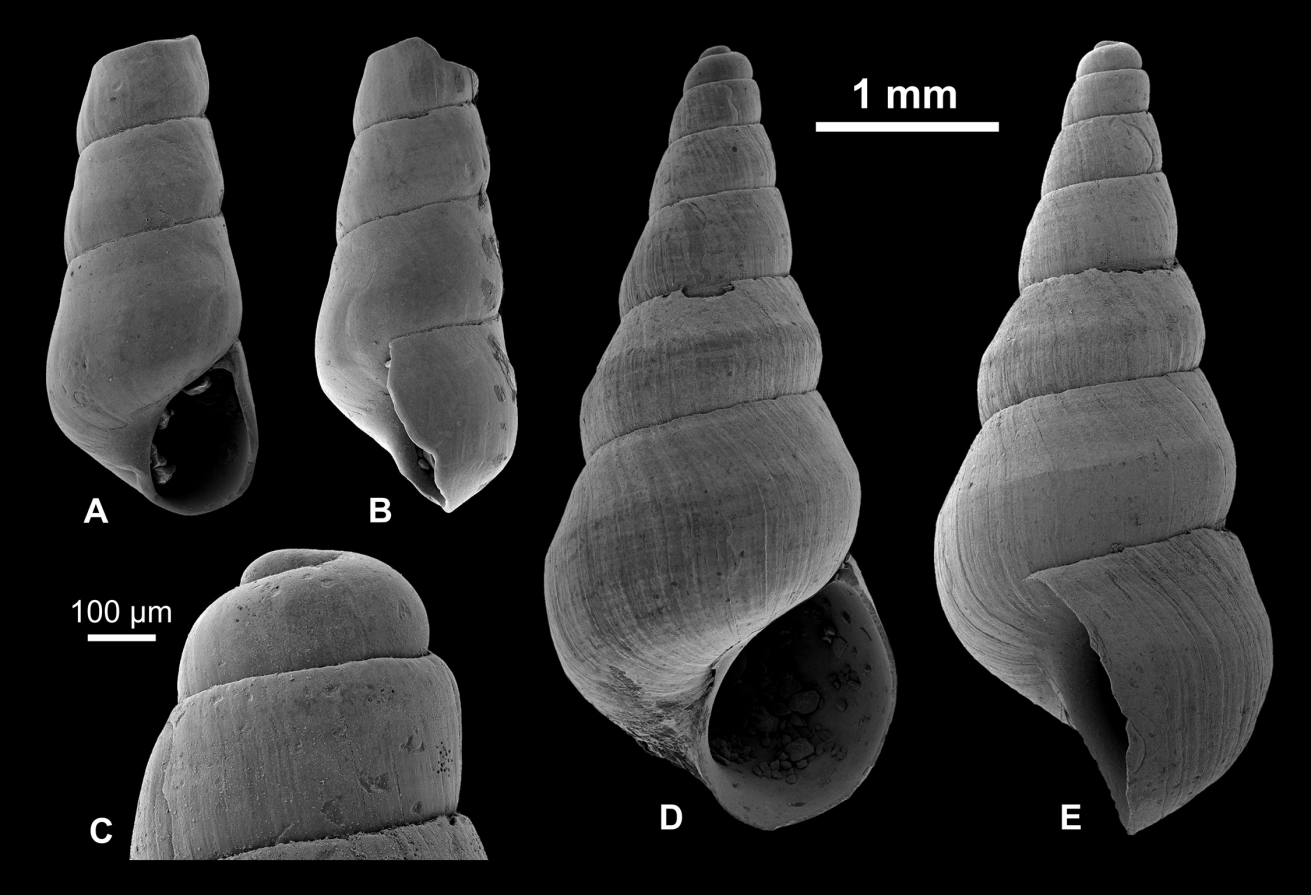
Pyrgulinae. **A–C** Turricaspia ? dimidiata (Eichwald, 1838), RGM 1309787 **D, E** *Turricaspia andrussowi* (B. Dybowski & Grochmalicki, 1915), RGM 1309814.

**Figure 12.**
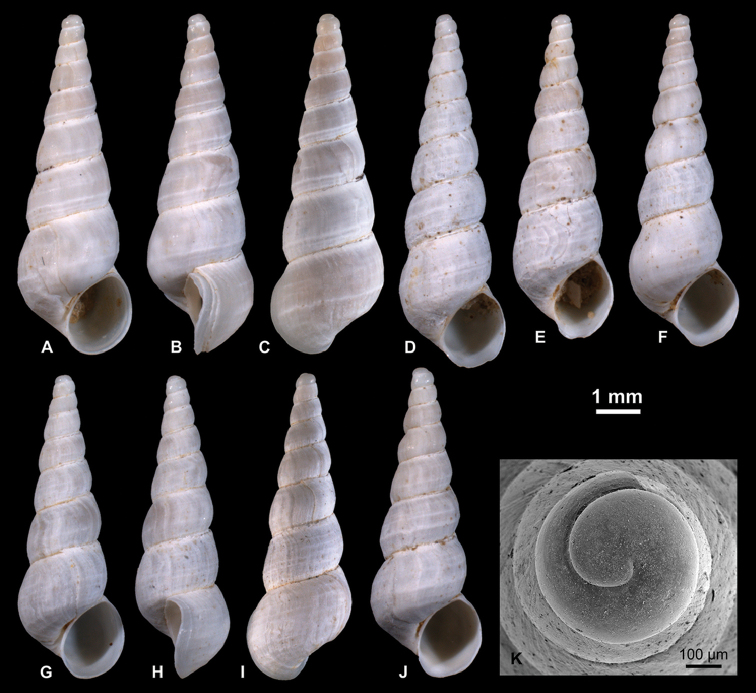
Pyrgulinae. **A–C** *Turricaspia lyrata* (B. Dybowski & Grochmalicki, 1915), RGM 1310213 **D** *T. lyrata*, RGM 1310220 **E** *T. lyrata*, RGM 1310214 **F** *T. lyrata*, LV 201512 **G–I** *T. lyrata*, LV 201513 **J** *T. lyrata*, RGM 1310218 **K** *T. lyrata*, RGM 1309802.

**Figure 13.**
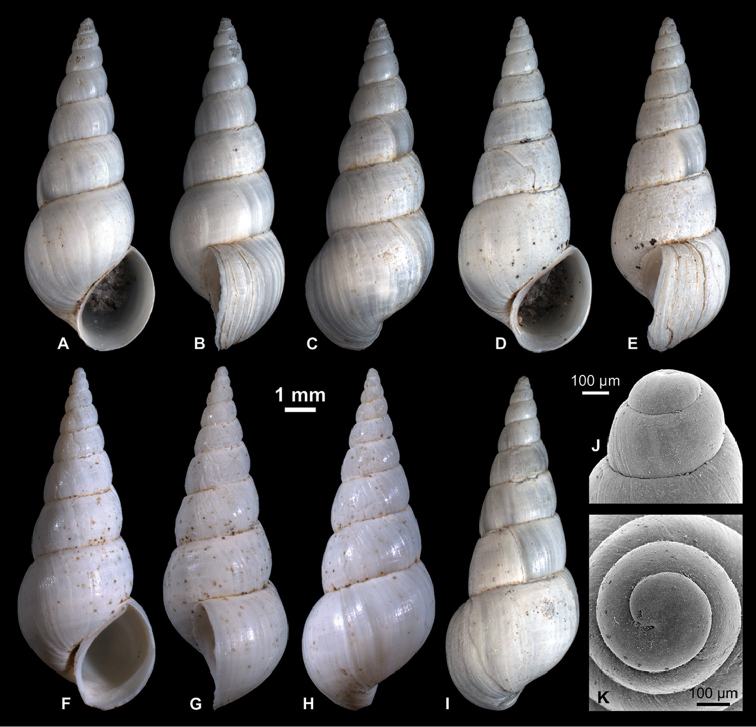
Pyrgulinae. **A–C** *Turricaspia meneghiniana* (Issel, 1865), RGM 1310256 **D, E, I** *T. meneghiniana*, LV 201518 **F–H** *T. meneghiniana*, RGM 1310197 **J, K** *T. meneghiniana*, RGM1309799.

**Figure 14.**
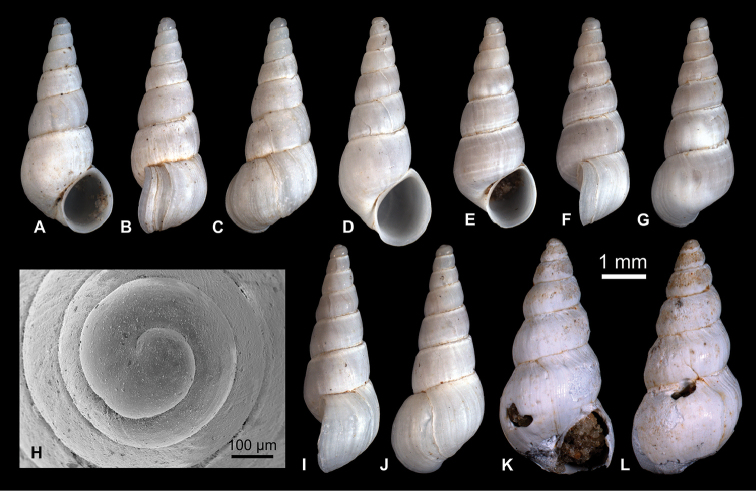
Pyrgulinae. **A–C** *Turricaspia pulla* (B. Dybowski & Grochmalicki, 1915), LV 201519 **D, I, J** *T. pulla*, RGM 1310254 **E–G** *T. pulla*, RGM 1310253 **H** *T. pulla*, RGM 1309820 **K, L** *Turricaspia pullula* (B. Dybowski & Grochmalicki, 1915), RGM 1310210.

*1838 Rissoa caspia m.; Eichwald: 154–155.
1841 Rissoa caspia – Eichwald: 256–257, pl. 38, figs 14–15.
1853 *Riss.*[*oa*] *caspia* m. – Eichwald: 273.
non 1876 Hydrobia caspia, Eichw. – Grimm: 150–153, pl. 6, fig. 15.
non 1877 Hydrobia caspia, Eichw. – Grimm: 79–80, pl. 7, figs 3a–d.
non 1887 Micromelania caspia Eichw. sp. – W. Dybowski: 21.
non 1888 *Micr.*[*omelania*] *caspia* Eichw. sp. – W. Dybowski: 78, pl. 1, fig. 1.
? 1896 *B.*[*uliminus*] (*Napaeus*?) *goebeli* Westerlund: 188.
1914 Micromelania (?) curta Nalivkin: 21–22, 31, pl. 6, figs 1–2 [partim; non figs 3–4, 7, 9–14].
1914 [Micromelania (?) curta] var. plano-convexa Nalivkin: 22, 31, pl. 6, figs 15–18.
non 1914 Micromelania caspia Eichw. – Nalivkin: 22, 31, pl. 6, figs 5–6 [partim; non fig. 8].
non 1917 Micromelania (Turricaspia, Laevicaspia) caspia Eichw. – B. Dybowski & Grochmalicki: 5–8, 36–38, pl. 1, figs 1–3.
non 1969 Pyrgula caspia (Eichw.). – Logvinenko & Starobogatov: 369–370, fig. 364 (1).
1987 *T.*[*urricaspia*] *caspia* (Eichw.). – Alexenko & Starobogatov: 33, fig. 2.
2006 Turricaspia caspia (Eichwald, 1838). – Kantor & Sysoev: 106, pl. 49, fig. M.
2014 Euxinipyrgula lincta. – Taviani et al.: 4, fig. 3c [non Micromelania lincta Milashevich, 1908].
2016 Turricaspia caspia (Eichwald, 1838). – Vinarski & Kantor: 246.

########## Material.

300 specimens (RGM 1309788, RGM 1309789, 1309797, RGM 1309798, RGM 1310196, RGM 1310257, RGM 1310258, LV 201511).

########## Type material.

Lectotype: ZIN (No. 1 in systematic catalogue), designated by Alexenko and Starobogatov (1987).

########## Type locality.

“In eodem lapide calcario Dagesthanico, fossilis” (in the same limestone of Dagestan [referring to the previous species, also found in Dagestan], fossil).

########## Dimensions.

9.01 × 3.31 mm (RGM 1310257, Fig. 8A–C); 7.88 × 3.31 mm (RGM 1310258, Fig. 8D–F); 10.33 × 3.92 mm (LV 201511, Fig. 8I–K); 9.92 × 3.83 mm; 10.21 × 3.88 mm; 9.52 × 3.54 mm; 9.69 × 3.61 mm.

########## Description.

Large, slender ovoid shell comprising up to 8.3 whorls. Protoconch large, measuring 535–600 µm at 1.15–1.2 whorls, with initial part inflated; nucleus almost immersed, 190–230 µm wide; nucleus and early protoconch bear intentions of malleate sculpture, which passes into granular surface after half a whorl accompanied by onset of spiral striae; P/T boundary indistinct. Whorl convexity decreasing rapidly: first teleoconch whorl moderately convex, second to last whorl low convex, sometimes almost straight-sided; maximum convexity is in lower half; whorls closely attached, suture narrow; a very small but marked convexity appears at upper suture, producing a faintly stepped spire; occasionally, it is accompanied by shallow abapical concavity. Last whorl makes up 46–50% of shell height, passing over regular but weakly convex to near straight-sided to slightly concave base. Aperture slender ovoid, inclined, closely attached to preceding whorl; in latest ontogeny, shell growth is more abapically directed, resulting in marked thickening at adapical tip. Peristome simple, thin, slightly expanded and indented at base; distinctly and regularly sigmoidal in lateral view, with upper half broadly indented and lower half broadly protruding; inner lip protrudes in lateral view, extending sheet-like over base of penultimate whorl; umbilicus mostly closed, rarely very narrow, slit-like. Growth lines weakly sigmoidal: strongly prosocline in upper half, weakly opisthocline in lower half. Several specimens show faint spiral threads on last and penultimate whorls.

########## Discussion.

Different concepts of this species previously applied have led to considerable confusion about its real identity. This is partly rooted in the description and illustration provided by Eichwald (1838, 1841) that were insufficient to allow safe discrimination from similar species. For instance, *Micromelania caspia* sensu Grimm (1876, 1877), W. Dybowski (1887–1888) and B. Dybowski and Grochmalicki (1917) differs from the present species in the acute apex, the moderately convex whorls, the deep suture and the thin peristome. As already noted by Alexenko and Starobogatov (1987), it represents a different species, i.e., *Laevicaspia lincta* (Milashevich, 1908). That species was described from Lake Katlabukh near the Danube delta in Ukraine (lectotype, which matches Milashevich’s description, is illustrated in Kantor and Sysoev 2006: 95, pl. 45, fig. D; as *Euxinipyrgula lincta*). Specimens from the Neoeuxinian (late Pleistocene) of the Marmara Sea identified as E. cf. lincta by Taviani et al. (2014) differ from that species in the near straight-sided whorls and thickened peristome; in fact, the material corresponds well to *L. caspia*.

*Micromelania caspia* sensu Nalivkin (1914) comprises at least two species, both being more elongate, having more whorls and relatively smaller last whorls than *L. caspia*. In turn, some of the illustrated syntypes of “*Micromelania*” *curta* Nalivkin, 1914 and the variety “*Micromelania*” *curta var. planoconvexa* Nalivkin, 1914 from Bakunian deposits of Shikhovo, Apsheron Peninsula, Azerbaijan, closely resemble the present species and are thus (partly) considered synonymous. “*Micromelania*” *curta* encompasses a great variability of shapes, ranging from slender, elongate (*caspia*-type) to broad, conical shells. Since no holotype or lectotype have been designated, the status of this species is unresolved at present. Note that *Pyrgula curta* sensu Logvinenko and Starobogatov (1969) and Kantor and Sysoev (2006) does not correspond to Nalivkin’s species but rather to the specimens Nalivkin (1914) misidentified as *Micromelania caspia*.

Similarly, *Pyrgula caspia* sensu Logvinenko and Starobogatov (1969) is a quite different species, showing highly convex whorls and an inflated last whorl. It rather ranges within the morphological variability of *Turricaspia meneghiniana* (see below).

Alexenko and Starobogatov (1987) finally brought stability to the identity of *L. caspia* by designating a lectotype (see Kantor and Sysoev 2006: 106, pl. 49, fig. A; as *Turricaspia caspia*), which matches well our specimens. The label accompanying their specimen reads “Kaspiyskoye more” (“Caspian Sea”), which differs from the information provided by Eichwald (Dagestan) (see also discussion in Vinarski and Kantor 2016: 246). Inspection of the catalogue of the Zoological Institute, Russian Academy of Sciences, St. Petersburg (ZIN), however, confirmed that the lectotype is based on Eichwald’s original material.

The similar *Laevicaspia iljinae* (Golikov & Starobogatov, 1966) from Holocene deposits of the Crimean Peninsula can be distinguished in its more slender shape and the spruce-like whorl outline (i.e., steep, straight-sided upper two-thirds passing over convexity into flatter, convex lower third; see also Kantor and Sysoev 2006: 108, pl. 49, fig. D).

########## Distribution.

Endemic to the Caspian Sea (Logvinenko and Starobogatov 1969 stated that the species occurs at a depth of 30–150 m in the middle and southern Caspian Sea, but these data have to be revised given their incorrect concept of *L. caspia*).

######### Laevicaspia cincta

Taxon classificationAnimaliaLittorinimorphaHydrobiidae

(Abich, 1859) comb. n.

Fig. 9A–H

*1859 Rissoa cincta; Abich: 57, pl. 2, fig. 6.
?1887 Caspia Orthii Clessin & W. Dybowski in W. Dybowski: 40.
?1888 [Caspia] Orthii n. sp. – W. Dybowski: 79, pl. 3, fig. 6.
1969 Pyrgula [(Caspiella)] cincta (Abich). – Logvinenko & Starobogatov: 372, fig. 366 (4).
2006 Pyrgula cincta (Abich, 1859). – Kantor & Sysoev: 98, pl. 47, fig. L.
2016 Pyrgula cincta (Abich, 1859). – Vinarski & Kantor: 236.

########## Material.

174 specimens (RGM 1309806, RGM 1309807, RGM 1310200, LV 201514).

########## Type material.

Not traced.

########## Type locality.

Abich (1859) specified the type locality on p. 12–13 as “Gulf of Baku”.

########## Dimensions.

3.83 × 1.93 mm (LV 201514, Fig. 9A, B); 4.05 × 1.89 mm (RGM 1309807, Fig. 9C–E); 4.41 × 2.10 mm (RGM 1309806, Fig. 9F–H); 4.73 × 2.11 mm; 4.59 × 2.10 mm; 4.58 × 2.17 mm.

########## Description.

Slender ovoid shell with up to 6.5 whorls. Protoconch broad, low dome-shaped, consists of 1.2 whorls that measure 415 µm in diameter, with slightly inflated initial part; nucleus 150 µm wide; protoconch surface weakly granulate, with intentions of striae on second half; P/T transition distinct, formed by sharp, thin axial line. Whorl convexity decreases steadily during ontogeny, with early teleoconch whorls being moderately convex and penultimate and last whorl low convex to almost straight-sided. On third teleoconch whorl, weak subsutural band emerges that slightly enhances during ontogeny; band forms weak bulge throughout, with maximum convexity in its lower half and steep, almost straight-sided ramp in upper half; abapical demarcation clear, sometimes accompanied by thin groove. Last whorl attains 54–65% of shell height, passing from flattened whorl flank over marked convexity into steep, straight-sided base. Aperture near drop-shaped, inclined, with acute adapical angle, straight parietal margin, obtuse angle between parietal and columellar margins, sometimes slightly expanded palatal margin. Peristome not thickened, weakly expanded at columella and base; regularly sinuate in lateral view, with broad adapical indentation and about equally broad and high abapical protrusion. Umbilicus closed or very narrow. Growth lines weakly prosocline in upper half, near orthocline in lower half.

########## Discussion.

The Selitrennoye specimens match with the original description in terms of size (shell height: 3–4 mm), the ovoid shell shape, the number of whorls, the rounded last whorl and the simple peristome margin; they differ in the expression of the subsutural band, which Abich indicated to be “weakly keeled”. We consider these differences to range within the intraspecific variability of this species.

*Laevicaspia cincta* can be readily distinguished from other Pontocaspian Pyrgulinae by its ovoid, slightly stepped shell with broad, blunt apex, subsutural band and flattened whorls in later ontogeny. *Laevicaspia abichi* (Logvinenko & Starobogatov, 1969) from the middle Caspian Sea, differs in the much larger size (6.8 × 3 mm), the conical shape, the narrower subsutural band and the larger aperture. The Caspian endemic species *Laevicaspia kowalewskii* (Clessin & W. Dybowski in W. Dybowski, 1887) resembles *L. cincta* very closely in terms of the slender ovoid shape with near straight-sided whorls, the closely attached aperture with thin peristome, and the lacking umbilicus; it differs in the lack of a subsutural band and the more elongate shape.

*Caspia orthii* Clessin & W. Dybowski in W. Dybowski, 1887 was synonymized with the present species by previous authors (e.g., Logvinenko and Starobogatov 1969, Kantor and Sysoev 2006, Vinarski and Kantor 2016). The original description matches well our specimens in terms of size (4.8 × 1.9 mm), number of whorls, expression of the subsutural band and shape of the aperture; the only difference is the “elongated-conical” shape compared to the ovoid shells of *L. cincta* described by Abich (1859) and represent by our material. Although not having seen W. Dybowski’s type material, we tentatively follow the previous assessment and consider *Caspia orthii* a junior synonym of *Laevicaspia cincta*.

Note that *Rissoa cincta* Deshayes, 1861 (p. 404, pl. 24, figs 4–6), described from the Eocene (Bartonian) of the Paris Basin, is a junior primary homonym of this species and thus invalid. At present, this species is classified in the genus *Pseudotaphrus* Cossmann, 1888 (Ponder 1984: 96).

########## Distribution.

Endemic to the Caspian Sea, in the southern part at a depth of >250 m (Parr et al. 2007).

######### Laevicaspia conus

Taxon classificationAnimaliaLittorinimorphaHydrobiidae

(Eichwald, 1838) comb. n.

Fig. 9I–P

*1838 Rissoa Conus m.; Eichwald: 155.
1841 Rissoa Conus m. – Eichwald: 257, pl. 38, figs 16a–b [wrongly given as “figs 16–17” on p. 257; see also corrigendum at the end of Eichwald’s work].
1853 *Riss.*[*oa*] *conus* m. – Eichwald: 273.
non 1876 Eulima conus, Eichw?. – Grimm: 154–156, pl. 6, fig. 14.
1887 Nematurella conus Eichw. sp. (non Grimm). – W. Dybowski: 45.
1888 [Nematurella] conus Eichw. sp. – W. Dybowski: 78, pl. 2, fig. 3.
? 1896 Prosostenia [sic] conus Eichw. – Sinzov: 49–50, pl. 1, figs 30–33.
1926 ?Nematurella conus (Eichwald). – Wenz: 2007.
1952 Caspiella conus (Eichwald, 1841). – Zhadin: 259, fig. 211.
1969 Pyrgula [(Caspiella)] conus (Eichw). – Logvinenko & Starobogatov: 374, fig. 366 (5–6).
non 2006 Turricaspia conus conus (Eichwald, 1838). – Kantor & Sysoev: 106, pl. 48, fig. J.
2016 Turricaspia conus conus (Eichwald, 1838). – Vinarski & Kantor: 246–247.

########## Material.

1135 specimens (RGM 1309828, RGM 1309829, RGM 1309830, RGM 1310199, RGM 1310226–1310228, LV 201515).

########## Type material.

Not traced.

########## Type locality.

“In eodem lapide calcareo, fossilis” (in the same limestone [referring to previous species, found in Dagestan], fossil).

########## Dimensions.

5.14 × 2.19 mm (RGM 1309830, Fig. 9I, J); 4.60 × 2.18 mm (LV 201515, Fig. 9K); 4.02 × 1.91 mm (RGM 1309829, Fig. 9L–O); 3.87 × 1.87 mm (RGM 1309828, Fig. 9P); 4.60 × 2.23 mm (RGM 1310226); 5.12 × 2.37 mm (RGM 1310227); 4.17 × 2.14 mm (RGM 1310228).

########## Description.

Ovoid, glossy shell with up to 6.8 whorls. Shell outline variable, depending on growth stage: shells with up to 5 whorls are rather broad, nearly conical; in late ontogeny, shell growth is directed adapically, producing more elongate shapes with narrow, high last whorl; sometimes, these slender elongate morphotypes have slightly irregular shape. Protoconch consists of 1.2 whorls with 355 µm in diameter; nucleus almost immersed, 125 µm wide; surface faintly malleate or granulate, with intentions of spiral sculpture detected in some specimens; P/T boundary very distinct, marked by sharp, thin axial line. Teleoconch whorls weakly to moderately convex, sometimes adapically flattened. Last whorl attains between 55–63% of total height, grades into straight-sided or weakly convex base. Aperture drop-shaped, inclined, closely attached to base of preceding whorl, usually covering or rarely leaving slit-like umbilicus. Peristome slightly expanded, thin or thickened all around, especially at adapical tip; regularly sinuate in lateral view, with broad adapical indentation and about equally broad and high abapical protrusion. Growth lines weak, prosocline in upper half, orthocline in lower half.

########## Discussion.

Logvinenko and Starobogatov (1969) listed “*Rissoa conus* Eichwald, 1841, partim” in synonymy of *Pyrgula kolesnikoviana* Logvinenko & Starobogatov in Golikov & Starobogatov, 1966 (now classified in *Laevicaspia*; see below), but without any explanation. The synonymy list was expanded as “*Rissoa conus* sensu Eichwald, 1841, partim, non Eichwald, 1838” by Kantor and Sysoev (2006) and Vinarski and Kantor (2016), yet again without discussion. The synonymy is not mentioned in the original description of *Laevicaspia kolesnikoviana* in Golikov and Starobogatov (1966). Very likely, the synonymy roots in the ambiguous description of Eichwald (1838, 1841), summarizing two different morphologies. Eichwald referred to the typical form as having a conical shell with seven, gently increasing whorls, whereas the last two are much broader; the size was indicated as 2 × 1 lin., which corresponds to 4.2 × 2.1 mm (given Eichwald used the Russian *liniya*). In addition, he mentioned rarer, slightly longer (3 lin.) specimens, with deeper suture and straight-sided whorls. In 1841, Eichwald illustrated one of these rare specimens. The description in the 1841-work, however, is almost identical to the original description. In this light, it remains unclear why Kantor and Sysoev (2006) and Vinarski and Kantor (2016) referred to as “*Rissoa conus* sensu Eichwald, 1841, partim, non Eichwald, 1838” in their synonymy lists of *L. kolesnikoviana*. To complete confusion, the specimen illustrated in Kantor and Sysoev (2006) is not *L. conus*, differing in the broad, blunt apex and the near straight-sided whorls; it rather resembles *L. kowalewskii* (Clessin & W. Dybowski in W. Dybowski, 1887).

The holotype of *L. kolesnikoviana* illustrated by Kantor and Sysoev (2006, pl. 47, fig. N) corresponds to the description and illustration of the rare, slender morphology of *Laevicaspia conus* sensu Eichwald in terms of the number of whorls and the near straight-sided whorls; it differs only in the considerably smaller size (3.7 mm vs. 6.3 mm). Yet, Golikov and Starobogatov (1966) and Logvinenko and Starobogatov (1969) indicate larger sizes for *L. kolesnikoviana* (5.5 mm and 6.5 mm, respectively), suggesting a great variability in size. On the other hand, the two morphologies delineated by Eichwald also match our own observations on *L. conus*. In late ontogeny, growth is directed almost entirely abapically, resulting in more elongate shells with an additional whorl. These larger morphologies correspond completely to the smaller, relatively bulkier shells in all other aspects, which is why we consider them as morphotypes rather than species-group taxa. Without Eichwald’s material at hand it is difficult to arrive at a conclusion on this matter.

The species has affinities with several representatives of the Azov and Black seas. Pyrgula (Caspiella) lindholmiana Golikov & Starobogatov, 1966, today considered as a subspecies of *L. conus* (e.g., Vinarski and Kantor 2016), has a larger and broader shell. Similarly, *Laevicaspia milachevitchi* (Golikov & Starobogatov, 1966) and *Laevicaspia boltovskoji* (Golikov & Starobogatov, 1966) are broader than *L. conus*, while *Laevicaspia lincta* (Milashevich, 1908) and *Laevicaspia limanica* (Golikov & Starobogatov, 1966) are more slender and larger.

“*Eulima conus* Eichwald” as described and illustrated by Grimm (1876, 1877) has little resemblance to actual *L. conus*. He illustrated a very elongate, conical shell with many more and almost perfectly straight-sided whorls. This fact was already noticed by Clessin and W. Dybowski a few years later, and they introduced *Micromelania grimmi* Clessin & W. Dybowski in W. Dybowski, 1887 for the misidentified species.

The illustrations of specimens from the Kuyalnikian (late Pliocene to early Pleistocene) of the Odessa region identified as *Prososthenia conus* by Sinzov (1896) show shells with similar shape, proportions and whorl convexity. A more detailed examination of material from the region is required to assess whether it is indeed conspecific with *L. conus*.

########## Distribution.

Endemic to the Caspian Sea, reported from depths between 0 and 120 m (Logvinenko and Starobogatov 1969).

######### Laevicaspia kolesnikoviana

Taxon classificationAnimaliaLittorinimorphaHydrobiidae

(Logvinenko & Starobogatov in Golikov & Starobogatov, 1966) comb. n.

Fig. 10A–E, K, N

*1966 *P.*[*yrgula*] (*Caspiella*) *kolesnikoviana* Logvinenko et Starobogatov; Golikov & Starobogatov: 357, fig. 2 (8–9).
1969 Pyrgula [(Caspiella)] kolesnikoviana Logv. et Star. – Logvinenko & Starobogatov: 372, fig. 366 (1).
2006 Pyrgula kolesnikoviana Logvinenko et Starobogatov in Golikov et Starobogatov, 1966. – Kantor & Sysoev: 100, pl. 47, fig. N.
2016 Pyrgula kolesnikoviana Logvinenko et Starobogatov in Golikov et Starobogatov, 1966. – Vinarski & Kantor: 239.

########## Material.

514 specimens (RGM 1309816, RGM 1309818, RGM 1309819, RGM 1310212, RGM 1310221–1310225, LV 201516).

########## Type material.

Holotype: ZIN 4462/1.

########## Type locality.

Caspian Sea, N of Apsheron peninsula, NW from Kamni Dva Brata Island, 40°47'N, 49°42'E, 30 m (Vinarski and Kantor 2016).

########## Dimensions.

3.55 × 1.63 mm (RGM 1309816, Fig. 10A–C, K); 3.59 × 1.67 mm (LV 201516, Fig. 10D); 3.95 × 1.82 mm (RGM 1309819, Fig. 10E, N); 3.90 × 1.77 mm (RGM 1309818); 4.04 × 1.96 mm (RGM 1310222); 4.49 × 1.99 mm (RGM 1310223); 3.54 × 1.72 mm (RGM 1310224).

########## Description.

Small, slender ovoid, shiny shell with up to 6.9 whorls. Protoconch consists of 1.2 whorls, measuring 355 µm in diameter; nucleus rather long, ca. 130 µm wide; surface finely granulate (maybe due to preservation; traces of finely malleate to irregularly striate pattern occurs on margins of nucleus and initial part); faint striae on last third; P/T boundary distinct. Whorl convexity of teleoconch whorls decreasing: first to second whorl moderately to highly convex, last whorl low to moderately convex. Faint subsutural band appears on later teleoconch whorls in some specimens, sometimes accompanied by weak concavity below. Last whorl attains 50–57% of shell height, passing via broad, regular convexity in to weakly convex base. Aperture ovoid, inclined, closely attached to preceding whorl; in latest ontogeny, shell growth is more abapically directed, resulting in marked thickening at adapical angle. Peristome thin or thickened all around, with parietal margin sometimes slightly expanded; weakly but regularly sinuate in lateral view, with broad adapical indentation and about equally broad and high abapical protrusion. Umbilicus usually closed or very narrow, slit-like. Growth lines weak, prosocline in upper half, orthocline in lower half. In addition, faint spiral furrows appear in some specimens.

########## Discussion.

Co-occurring *Laevicaspia vinarskii* sp. n. differs in the consistently lower whorl expansion rate at the same size and the smaller aperture. *Laevicaspia kowalewskii* (Clessin & W. Dybowski in W. Dybowski, 1887) can be distinguished by its broader and larger shell.

########## Distribution.

Endemic to the Caspian Sea, reported from depths between 25 and 180 m (Kolesnikov 1947, Logvinenko and Starobogatov 1969).

######### Laevicaspia vinarskii sp. n.

Taxon classificationAnimaliaLittorinimorphaHydrobiidae

http://zoobank.org/8399A902-945D-444A-A8AD-136592F8E527

Fig. 10F–J, L–M, O

########## Type material.

Holotype: LV 201517; 3.70 × 1.72 mm (Fig. 10F–G). Paratypes: RGM 1309821; 3.34 × 1.48 mm (Fig. 10I, J, L, M). RGM 1309805; 3.61 × 1.54 mm (Fig. 10H, O). LV 201731; 4.14 × 1.93 mm.

########## Additional material.

5 specimens (RGM 1309793, LV 201732).

########## Type locality.

Selitrennoye, Astrakhan, Russia; northern Caspian Basin; GPS coordinates: 47°10'21.19"N, 47°26'25.41"E (WGS84).

########## Age.

Early Late Pleistocene (late Khazarian, MIS 5).

########## Etymology.

In honor of Maxim Vinarski (Saint Petersburg State University) for his contributions to Malacology.

########## Diagnosis.

Slender ovoid, imperforate shell with up to 6.5 moderately convex whorls, narrow suture, granulate–striate protoconch, high whorl expansion rate and small, adnate, inclined aperture.

########## Description.

Slender ovoid shell with up to 6.5 whorls. Protoconch consists of 1.2 whorls measuring 375 µm; nucleus is 140 µm wide; surface strongly granulate on nucleus, less so on remaining protoconch, striae appear on last 0.25 whorls; P/T transition marked by distinct growth rim. Teleoconch whorls moderately convex, separated by narrow suture; whorls increase slowly in height, with the last attaining 53–57% of shell height, passing into weakly convex base. Weak subsutural band is observed in one specimen. Aperture small, inclined, closely attached to base of preceding whorl, leaving no or slit-like umbilicus. Peristome slightly thickened, especially at adapical tip; regularly sinuate in lateral view, with broad adapical indentation and about equally broad and high abapical protrusion. Distinct spiral furrows occur in well preserved specimens. Growth lines weak, prosocline in upper half, orthocline in lower half.

########## Discussion.

The new species differs from co-occurring *Laevicaspia kolesnikoviana* in the higher whorl expansion rate at about the same size and the larger aperture. Laevicaspia ? ismailensis (Golikov & Starobogatov, 1966) from lakes Yalpug and Kugurlu in the Danube river delta is more slender and larger (5.6 mm) at the same number of whorls and has a less inclined, rounder aperture (see holotype illustrated by Kantor and Sysoev 2006: pl. 50, fig. A).

########## Distribution.

Endemic to the Caspian Sea Pleistocene, so far only known from Selitrennoye.

######### Genus Turricaspia

Taxon classificationAnimaliaLittorinimorphaHydrobiidae

B. Dybowski & Grochmalicki, 1915

1915 Turricaspia B. Dybowski & Grochmalicki: 105.
1917 Trachycaspia B. Dybowski & Grochmalicki: 22.
1969 Pyrgula (Caspiopyrgula) Logvinenko & Starobogatov: 366.
1969 Pyrgula (Eurycaspia) Logvinenko & Starobogatov: 357.
1969 Pyrgula (Oxypyrgula) Logvinenko & Starobogatov: 366.

########## Type species.

*Micromelania turricula* B. Dybowski & Grochmalicki, 1915; by subsequent designation by Wenz (1939). Caspian Sea, Recent.

######### Turricaspia andrussowi

Taxon classificationAnimaliaLittorinimorphaHydrobiidae

(B. Dybowski & Grochmalicki, 1915)

Fig. 11A, B

*1915 Micromelania (Turricaspia) Andrussowi nov. sp.; B. Dybowski & Grochmalicki: 125–126, pl. 3, figs 31a–b.
1917 Micromelania (Turricaspia, Trachycaspia) Andrussowi nov. sp. – B. Dybowski & Grochmalicki: 26–27, pl. 4, fig. 39.
1969 Pyrgula [(Turricaspia)] andrusovi [sic] (Dyb. et Gr.). – Logvinenko & Starobogatov: 365–366, fig. 362 (4) [partim].
2006 Turricaspia andrussowi (B. Dybowski et Grochmalicki, 1915). – Kantor & Sysoev: 104–105, pl. 48, fig. A [partim].
2016 Turricaspia andrussowi (B. Dybowski et Grochmalicki, 1915). – Vinarski & Kantor: 245 [partim].

########## Material.

3 spire fragments (RGM 1309814, RGM 1310205).

########## Type material.

Lectotype: ZIN 4355/1 (specimen illustrated by B. Dybowski and Grochmalicki 1915, 1917), designated by Logvinenko and Starobogatov (1969), illustrated by Kantor and Sysoev (2006: pl. 48, fig. A).

########## Type locality.

Caspian Sea (no locality specified).

########## Description.

Available fragments indicate very slender, conical shell. Apex broad, blunt, bulbous. Whorl profile flattened, very weakly spruce-like, with straight-sided upper two-thirds passing over convexity into weakly convex lower third; in addition, broad, flat subsutural band appears, sometimes accompanied by very narrow concavity below. Umbilicus seems fully closed. Aperture not preserved in any specimen.

########## Discussion.

The identification of the three spire fragments rests upon the strongly adpressed whorls with very narrow suture and the flattened, spruce-like whorl profile, and the large, bulbous protoconch. *Turricaspia eulimellula* (B. Dybowski & Grochmalicki, 1915) has a similarly slender spire with adpressed whorls, but it bears a basal keel and the maximum whorl convexity is around mid-height instead of in the lower third. *Turricaspia grimmi* (Clessin & W. Dybowski in W. Dybowski, 1887) differs in its perfectly straight-sided, rectangular, very weakly stepped whorl profile (see also B. Dybowski and Grochmalicki 1917, pl. 3, figs 34–35; Kantor and Sysoev 2006, pl. 46, fig. L).

A very similar species is *Pyrgula dubia* Logvinenko & Starobogatov, 1969 from the middle Caspian Sea, matching the present one in the weakly spruce-like whorl profile; in fact, it might just be a juvenile specimen of *T. andrussowi*. Similarly, *Pyrgula turkmenica* Logvinenko & Starobogatov, 1969, from the eastern part of southern Caspian Sea, corresponds to *T. andrussowi* in the weak subsutural band accompanied by an abapical concavity; it might as well be a juvenile representative of *T. andrussowi*.

Logvinenko and Starobogatov (1969) synonymized without discussion *Hydrobia spica* sensu Grimm, 1876, *Turricaspia elegantula* sensu B. Dybowski & Grochmalicki, 1915, *T. brusinae* (B. Dybowski & Grochmalicki, 1915), as well as several varieties of *T. spica* and *T. turricula* described by B. Dybowski & Grochmalicki (1915), with *T. andrussowi* (see also Kantor and Sysoev 2006, Vinarski and Kantor 2016). However, none of these taxa actually resembles *T. andrussowi*. This species can be well delimited from these alleged synonyms in its bulbous protoconch and the characteristic, weakly spruce-like whorl profile. (Note that the drawing provided by Logvinenko and Starobogatov 1969 shows a rather broad shell with acute apex; it has little in common with the lectotype designated by them).

########## Distribution.

Endemic to the Caspian Sea (Logvinenko and Starobogatov 1969 indicated occurrences for the middle and southern Caspian Sea at depths of 25–80 m, but based on a much wider concept of the species).

######### Turricaspia ? dimidiata

Taxon classificationAnimaliaLittorinimorphaHydrobiidae

(Eichwald, 1838)

Fig. 11C–E

?*1838 Rissoa dimidiata m.; Eichwald: 156.
? 1841 Rissoa dimidiata m. – Eichwald: 258, pl. 38, figs 17a–b [wrongly given as “figs 16–17” on p. 258; see also corrigendum at the end of Eichwald’s work].
? 1853 Pal.[*udina*] dimidiata m. – Eichwald: 285–286.
? 1887 Micromelania dimidiata Eichw. sp. – W. Dybowski: 31 [partim].
? 1888 Micromelania dimidiata Eichw. sp. – W. Dybowski: 78, pl. 1, figs 4a–f, 5 [partim].
? 1917 Micromelania (Turricaspia) dimidiata Eichw. – B. Dybowski & Grochmalicki: 32–33, pl. 4, figs 44–47 [partim].
? 1969 Pyrgula dimidiata (Eichw.). – Logvinenko & Starobogatov: 358–359, fig. 359 (1).
? 2006 Pyrgula dimidiata (Eichwald, 1838). – Kantor & Sysoev: 99, pl. 46, fig. K.
? 2016 Pyrgula dimidiata (Eichwald, 1838). – Vinarski & Kantor: 238.

########## Material.

1 subadult specimen (RGM 1309787).

########## Type material.

Not traced.

########## Type locality.

“In eodem lapide calcareo, fossilis” (in the same limestone [referring to the previous species, found in Dagestan], fossil).

########## Dimensions.

4.29 × 1.93 mm.

########## Description.

Slender elongate shell with ca. 6.5 whorls preserved. Protoconch granulate, originally perhaps densely malleate. First teleoconch whorl straight-sided in profile, passing into weakly convex outline on 2^nd^–3^rd^ whorl. Between 3^rd^ and 4^th^ whorl, broad, blunt central swelling emerges, grading into thin angulation on 5^th^ whorl; no keel is developed. Whorl portion above swelling/angulation straight-sided, below weakly convex; directly above it, weak concavity is formed locally. Aperture ovoid, strongly adnate, leaving no umbilicus, with thin peristome. Growth lines rather distinct, with prosocline upper half and near orthocline lower half.

########## Discussion.

A single subadult shell containing ca. 6.5 whorls (including the protoconch) is available. Size and number of whorls as well as the centrally placed angulation correspond well to Eichwald’s (1838, 1841) description and illustration of *T. dimidiata*. However, the central keel is very weakly expressed in our specimen and it starts not before the fourth whorl, which is why we only tentatively attribute it to this species.

Kantor and Sysoev (2006) illustrate a much more elongate specimen with cyrtoconoid spire and more abapically placed keel; it might represent a different species. *Turricaspia bakuana* (Kolesnikov, 1947), likewise described from Caspian Sea, too has a central keel, but differs in the much more slender shell and consistently strong keel from the second teleoconch whorl onwards (cf. Kantor and Sysoev 2006). *Turricaspia basalis* (B. Dybowski & Grochmalicki, 1915) has a broader conical habitus and the keel is placed near the lower suture. The subspecies *T. b. laticarinata* (Logvinenko & Starobogatov, 1969) only differs from *T. basalis* in the thickness of the keel and is herein considered a junior synonym of the nominal species.

########## Distribution.

Endemic to the Caspian Sea, reported from middle and south Caspian Sea at depths between 35 and 200 m (Logvinenko and Starobogatov 1969).

######### Turricaspia lyrata

Taxon classificationAnimaliaLittorinimorphaHydrobiidae

(B. Dybowski & Grochmalicki, 1915)

Fig. 12A–K

*1915 Micromelania (Turricaspia) spica Eichw. var. lyrata nov. var.; B. Dybowski & Grochmalicki: 117, pl. 2, fig. 18.
1915 Micromelania (Turricaspia) spica Eichw. var. incisata nov. var.; B. Dybowski & Grochmalicki: 117, pl. 2, fig. 19.
1915 Micromelania (Turricaspia) spica Eichw. var. striata nov. var.; B. Dybowski & Grochmalicki: 117, pl. 2, fig. 20.
1917 Micromelania (Turricaspia) spica Eichw. var. lyrata nov. var. – B. Dybowski & Grochmalicki: 17, pl. 3, fig. 25.
1917 Micromelania (Turricaspia) spica Eichw. var. incisata nov. var. – B. Dybowski & Grochmalicki: 18, pl. 3, fig. 26.
1917 Micromelania (Turricaspia) spica Eichw. var. striata nov. var. – B. Dybowski & Grochmalicki: 18, pl. 3, fig. 27.
1969 Pyrgula [(Turricaspia)] lirata [sic] (Dyb. et Gr.). – Logvinenko & Starobogatov: 365, fig. 362 (2).
2006 Pyrgula lirata [sic] (B. Dybowski et Grochmalicki, 1915). – Kantor & Sysoev: 101, pl. 46, fig. E.
2016 Pyrgula lirata [sic] (B. Dybowski et Grochmalicki, 1915). – Vinarski & Kantor: 240.

########## Material.

562 specimens (RGM 1309802, RGM 1309825, RGM 1310209, RGM 1310213, RGM 1310214, RGM 1310216, RGM 1310218–1310220, LV 201512, LV 201513).

########## Type material.

Lectotype: ZIN 4552/1 (specimen illustrated by B. Dybowski and Grochmalicki 1915, 1917), designated by Logvinenko and Starobogatov (1969), illustrated by Kantor and Sysoev (2006: pl. 46, fig. E).

########## Type locality.

Caspian Sea (no locality specified).

########## Dimensions.

7.68 × 2.59 mm (RGM 1310213, Fig. 12A–C); 7.99 × 2.42 mm (RGM 1310220, Fig. 12D); 7.34 × 2.28 mm (RGM 1310214, Fig. 12E); 7.54 × 2.50 mm (LV 201512, Fig. 12F); 6.87 × 2.43 mm (LV 201513, Fig. 12G–I); 7 × 2.52 mm (RGM 1310218, Fig. 12J).

########## Description.

Slender elongate shell of up to 9 whorls. Protoconch large, measuring about 485 µm in diameter; it forms bulbous cap on top of shell and comprises 1.25 whorls; surface weakly granulate, with striae on last 0.25 whorls; nucleus low, broad, ca. 170 µm in diameter; P/T transition very distinct, marked by sharp growth cessation. Teleoconch whorls low to moderately convex, often flattened or with straight-sided upper half, which creates spruce-like morphology. Sometimes, very weak and thin bulge appears below suture, producing faintly stepped spire. Most shells bear very low and somewhat irregular spirals, but expression varies considerably concerning its onset (mainly starts on lower whorls), strength (faint traces to distinct but blunt keels) and number of elements (one keel near base to several keels spread across whorl profile). Expression of sculpture varies in most specimens throughout ontogeny, which creates uneven, rugged appearance. Aperture comparatively small, in most cases regularly ovoid and weakly inclined, covering up umbilicus entirely or leaving very thin opening; peristome simple. Growth lines strongly sigmoidal, with prosocline upper third and opisthocline lower two-thirds.

########## Discussion.

This species can be distinguished from its congeners in its large, bulbous protoconch and the typical, somewhat irregular sculpture. It is consistently larger, more massive and on average bears much stronger sculpture than co-occurring T. ? spica. The varieties “Micromelania (Turricaspia) spica var. incisata” and “M. (T.) spica var. striata” introduced by B. Dybowski and Grochmalicki (1915) only differ in the depth of the suture and the expression of the teleoconch sculpture, respectively. Given the variability of these features, we consider both of them synonymous with *T. lyrata*. Already Logvinenko and Starobogatov (1969) considered *incisata* and *lyrata* synonymous and, as first revisers, chose *lyrata* as the valid name of the species. The variety “M. (T.) spica var. lLittorinimorphasa” B. Dybowski & Grochmalicki, 1915 might also be a synonym of this species. However, the apex of the specimen illustrated in B. Dybowski and Grochmalicki (1915, 1917), which contains diagnostic characters, is not preserved. Nevertheless, *striata* and *lLittorinimorphasa* are certainly not synonymous with *T. andrussowi* as suggested by Logvinenko and Starobogatov (1969). That species differs from *T. lyrata* in the much slender whorls with spruce-like, near straight-sided profile.

########## Distribution.

Endemic to the Caspian Sea (after Logvinenko and Starobogatov 1969, it occurs in the western part of the middle and southern Caspian Sea at a depth of 25–50 m; mind however that these authors used a slightly different concept of the species).

######### Turricaspia meneghiniana

Taxon classificationAnimaliaLittorinimorphaHydrobiidae

(Issel, 1865)

Fig. 13A–K

*1865 *Bythinia Meneghiniana*, Issel; Issel: 21, pl. 1, figs 12–13.
1866 *Bythinia Meneghiniana*, Issel. – Issel: 405, pl. 1, figs 12–13.
1917 Micromelania (Turricaspia) caspia Eichw. var. inflata nov. var. – B. Dybowski & Grochmalicki: 9, pl. 1, fig. 5.
? 1969 Pyrgula caspia (Eichw). – Logvinenko & Starobogatov: 369–370, fig. 364 (1).
? 1969 Pyrgula meneghiniana (Issel). – Logvinenko & Starobogatov: 370, fig. 365 (2).
non 1987 *T.*[*urricaspia*] *meneghiniana meneghiniana* (Iss.). – Alexenko & Starobogatov: 35, fig. 8.
2006 Turricaspia meneghiniana (Issel, 1865). – Kantor & Sysoev: 109, pl. 49, fig. E.
2016 Turricaspia meneghiniana (Issel, 1865). – Vinarski & Kantor: 248.

########## Material.

248 specimens (RGM 1309799, RGM 1309800, RGM 1310197, RGM 1310198, RGM 1310256, LV 201518).

########## Type material.

Not traced.

########## Type locality.

“Nei giacimenti fossiliferi di Baku” (from fossil deposits in Baku).

########## Dimensions.

10.86 × 4.27 mm (RGM 1310256, Fig. 13A–C); 10.91 × 4.36 mm (LV 201518, Fig. 13D, E, I); 11.17 × 4.50 mm (RGM 1310197, Fig. 13F–H); 10.82 × 4.14 mm; 11.23 × 4.40 mm; 11.65 × 4.49 mm.

########## Description.

Conical shell with up to 9.3 whorls. Protoconch comprises 1.3 whorls, measuring 440 µm in diameter, with slightly inflated initial part; nucleus measures 150 µm in diameter; entire protoconch surface weakly granulate; indistinct spiral striae appear on second half; P/T transition distinct, formed by sharp, thin axial line. Teleoconch whorls increase slowly but regularly in height and width; whorls moderately convex, whereas convexity slightly decreases with ontogeny. Last whorl attains 45–48% of shell height, passes over perfect convexity into slightly convex base. Aperture ovoid, inclined, closely attached to base of preceding whorl across almost entire parietal margin. Peristome thin, not thickened, little expanded; weakly sigmoidal in lateral view, with broad, shallow indentation in upper half and broad, weak protrusion in lower half; inner lip protrudes in lateral view, extending sheet-like over base of penultimate whorl; umbilicus very narrow, slit-like. Growth lines weakly sigmoidal: strongly prosocline in upper half, weakly opisthocline in lower half. Several specimens show faint spiral threads on last and penultimate whorls.

########## Discussion.

Our material matches well to the description of Issel (1865), corresponding in the conical shell shape, the regularly increasing whorls, the rounded last whorl with faint spiral striae, and the ovate, adapically angulated aperture; only his specimens (13.5 × 5 mm) are larger than ours and consist of more whorls. Compared to his description, Issel’s illustration seem to overemphasize the relative height of the last whorl and underrepresent the pronounced whorl convexity. However, variability as to these characteristics is discernible also in our material.

*Micromelania subulata* Westerlund, 1902 is commonly listed as junior synonym of this species but always without discussion (e.g., Logvinenko and Starobogatov 1969, Kantor and Sysoev 2006, Vinarski and Kantor 2016). Westerlund’s (1902b) description refers to a large (15 mm), elongate shell with 9.5–10 whorls and a thickened callus connecting the peristome margins. These features partly oppose Issel’s description, which is why we tend to consider both taxa as separate, in contrast to most previous authors. Unfortunately, Westerlund’s (1902b) type material of this species could not be traced, neither in the Göteborg Natural History Museum nor the Swedish Museum of Natural History in Stockholm, where the largest part of Westerlund’s material is stored (Vinarski et al. 2013).

Another commonly cited synonym is Micromelania caspia var. inflata B. Dybowski & Grochmalicki, 1915, which indeed matches both Issel’s description and our material.

*Turricaspia meneghiniana* differs from the similarly large *Laevicaspia caspia* (Eichwald, 1838) in its regularly conical profile, the higher number of whorls, and the higher whorl convexity. The drawings of “*Pyrgula meneghiniana* (Issel)” provided by Logvinenko and Starobogatov (1969) indicate a broader shell with low whorl convexity and might represent a different species. In contrast, *Pyrgula caspia* sensu Logvinenko and Starobogatov (1969) (non Eichwald 1838) resembles the present species in terms of the high shell convexity and regular growth rate and might be conspecific. *Turricaspia meneghiniana* sensu Alexenko and Starobogatov (1987), with few, low convex whorls and an angled base, is clearly a different species.

########## Distribution.

Endemic to the Caspian Sea, reported from middle and south Caspian Sea at depths between 0 and 35 m (Logvinenko and Starobogatov 1969).

######### Turricaspia pulla

Taxon classificationAnimaliaLittorinimorphaHydrobiidae

(B. Dybowski & Grochmalicki, 1915)

Fig. 14A–J

*1915 Micromelania (Turricaspia) caspia Eichw. var. pulla nov. var.; B. Dybowski & Grochmalicki: 111, pl. 1, fig. 6a.
1917 Micromelania (Turricaspia) caspia Eichw. var. pulla nov. var. – B. Dybowski & Grochmalicki: 10, pl. 1, fig. 7.
1969 Pyrgula [(Turricaspia)] pulla (Dyb. et Gr.). – Logvinenko & Starobogatov: 361–362, fig. 360 (8).
2006 Pyrgula pulla (B. Dybowski et Grochmalicki, 1915). – Kantor & Sysoev: 102, pl. 46, fig. C.
2016 Pyrgula pulla (B. Dybowski et Grochmalicki, 1915). – Vinarski & Kantor: 242.

########## Material.

186 specimens (RGM 1309803, RGM 1309804, RGM 1309820, RGM 1310211, RGM 1310253–1310254, LV 201519).

########## Type material.

Lectotype: ZIN 4422/1 (specimen illustrated by B. Dybowski and Grochmalicki 1915, 1917), designated by Logvinenko and Starobogatov (1969), illustrated by Kantor and Sysoev (2006: pl. 46, fig. C).

########## Type locality.

Caspian Sea (no locality specified).

########## Dimensions.

4.88 × 2.10 mm (LV 201519, Fig. 14A–C); 5.17 × 2.11 mm (RGM 1310254, Fig. 14D, I, J); 4.77 × 1.90 mm (RGM 1310253, Fig. 14E–G); 5.84 × 2.16 mm (RGM 1309803); 5.54 × 1.97 mm (RGM 1309804).

########## Description.

Slender conical shell with up to 8 whorls. Protoconch bulbous, weakly granulate, with striae on second half; diameter 410 µm, consists of 1.25 whorls; nucleus low, broad, 140 µm wide; transition to teleoconch distinct. Teleoconch whorls weakly convex, with maximum convexity at or slightly below midline of whorl profile; portion above maximum convexity almost straight-sided, portion below weakly convex. Whorls are separated by deep suture. Height of last whorl amounts 45% of total shell. Sometimes intentions of spiral lines appear on lower half of last whorl. Aperture ovoid, oblique, with weakly thickened and slightly expanded peristome; in lateral view, peristome is distinctly sigmoidal, with broad, shallow indentation in upper half and broad, weak protrusion in lower half. Umbilicus very narrow or closed. Growth lines sigmoidal, markedly prosocline in upper half, weakly opisthocline in lower half.

########## Discussion.

The species can be easily distinguished from most other species of *Turricaspia* by its comparably broad conical shape, the low-convex whorls, and its small size. Juvenile specimens of *T. meneghiniana* remind of *T. pulla* but the former have broader shells with more convex whorls. *Turricaspia pullula* is likewise broader and exposes a characteristic tripartite whorl profile (see below).

########## Distribution.

Endemic to the Caspian Sea, reported from middle and south Caspian Sea at depths between 15 and 75 m (Logvinenko and Starobogatov 1969).

######### Turricaspia pullula

Taxon classificationAnimaliaLittorinimorphaHydrobiidae

(B. Dybowski & Grochmalicki, 1915)

Fig. 14K, L

*1915 Micromelania (Turricaspia) caspia Eichw. var. pullula nov. var.; B. Dybowski & Grochmalicki: 111–112, pl. 1, fig. 7.
1917 Micromelania (Turricaspia) caspia Eichw. var. pullula nov. var. – B. Dybowski & Grochmalicki: 10–11, pl. 1, fig. 8.
1969 Pyrgula [(Turricaspia)] pullula (Dyb. et Gr.). – Logvinenko & Starobogatov: 366–367, fig. 363 (3).
2006 Turricaspia pullula (B. Dybowski et Grochmalicki, 1915). – Kantor & Sysoev: 109, pl. 50, fig. B.
2016 Turricaspia pullula (B. Dybowski et Grochmalicki, 1915). – Vinarski & Kantor: 249.

########## Material.

1 damaged specimen (RGM 1310210).

########## Type material.

Lectotype: ZIN 4423/1 (specimen illustrated by B. Dybowski and Grochmalicki 1915, 1917), designated by Logvinenko and Starobogatov (1969), illustrated by Kantor and Sysoev (2006: pl. 50, fig. B).

########## Type locality.

Caspian Sea (no locality specified).

########## Dimensions.

5.36 × 2.62 mm.

########## Description.

A single incomplete specimen of about 6 whorls is preserved. Protoconch is corroded beyond recognition. Early teleoconch whorls are poorly convex to centrally flattened. Convexity strongly increases on about 3^rd^ whorl. From 4^th^ whorl onwards, whorl surface is partitioned into three zones: two lower zones are roughly straight-sided in profile, upper one slightly concave; middle zone slightly wider than other two; zones are separated by blunt angulations, whose expression varies between very faint to distinct (but no keel is formed). Aperture not preserved, but the tight coiling of the last preserved whorl suggests that umbilicus is absent. Growth lines strongly prosocline in upper third, near orthocline in lower two-thirds; transition coincides with boundary between upper and middle zone.

########## Discussion.

The available specimen corresponds well to the lectotype as illustrated by Kantor and Sysoev (2006). The very characteristic tripartite whorl profile is only discernible on the penultimate whorl of their specimen. Such a pattern is unknown for any other Pontocaspian Pyrgulinae.

########## Distribution.

Endemic to the Caspian Sea, reported from the western part of the middle Caspian Sea at a depth of 60 m (Logvinenko and Starobogatov 1969).

######### Turricaspia ? spica

Taxon classificationAnimaliaLittorinimorphaHydrobiidae

(Eichwald, 1855)

Fig. 15A–R

? *1855 Paludina spica m.; Eichwald: 303–304, pl. 10, figs 8–9.
? 1887 Micromelania spica Eichw. sp. – W. Dybowski: 29–31.
? 1888 *Micr.*[*omelania*] *spica* Eichw. sp. – W. Dybowski: 78, pl. 1, figs 6a–c, pl. 3, figs 11a–d.
? 1917 Micromelania (Turricaspia) spica Eichw. – B. Dybowski & Grochmalicki: 16–17, pl. 3, figs 22–27.
? 1952 Micromelania spica (Eichwald, 1855). – Zhadin: 252–253, fig. 194.
? 1992 Turricaspia spica. – Anistratenko & Prisyazhniuk: 19, fig. 2d.
? 2006 Turricaspia spica (Eichwald, 1855). – Kantor & Sysoev: 110, pl. 49, fig. F.
? 2009 Turricaspia cf. spica (Eichwald, 1855). – Filippov & Riedel: 70, 72, 74, 76, figs 4e–f.
? 2016 Turricaspia spica (Eichwald, 1855). – Vinarski & Kantor: 250.

########## Material.

1420 specimens (RGM 1309784, RGM 1309785, RGM 1309786, RGM 1309811, RGM 1309812, RGM 1309813, RGM 1310229–1310231, RGM 1310233–1310237, RGM 1310239, RGM 1310240, LV 201501, LV 201502).

########## Type material.

Not traced, most probably in ZIN (Vinarski and Kantor 2016).

########## Type locality.

“Im kapischen Meere, am Ufer der Insel Tschetschnja, vorzüglich nLittorinimorphastwärs von der Insel im Meeresgrunde” (in the Caspian Sea, at the shores of Ostrov Chechen’, especially on the seafloor northeast of the island).

########## Dimensions.

**Figure 15.**
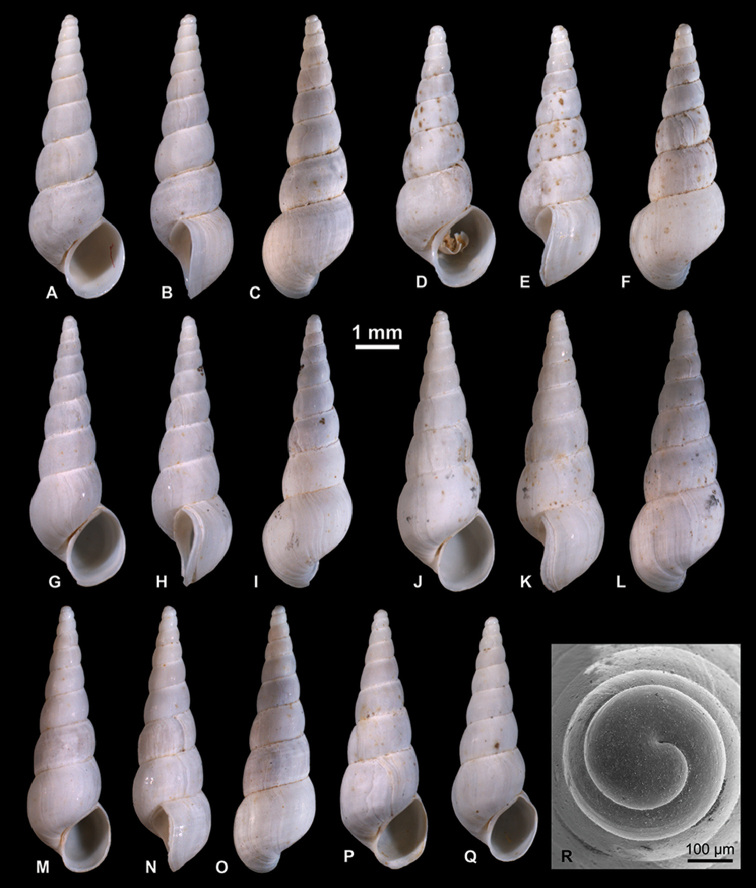
Pyrgulinae. **A–C** Turricaspia ? spica (Eichwald, 1855), form B, RGM 1310237 **D–F** T. ? spica, transitional form, LV 201501 **G–I** T. ? spica, form B, LV 201502 **J–L** T. ? spica, form A, RGM 1310230 **M–O** T. ? spica, form B, RGM 1310231 **P** T. ? spica, form A, RGM 1310233 **Q** T. ? spica, transitional form, RGM 1310236 **R** T. ? spica, RGM 1309813.

6.40 × 2.18 mm (RGM 1310237, Fig. 15A–C); 5.93 × 2.27 mm (LV 201501, Fig. 15D–F); 6.13 × 2.19 mm (LV 201502, Fig. 15G–I); 6.36 × 2.21 mm (RGM 1310230, Fig. 15J–L); 6.01 × 1.90 mm (RGM 1310231, Fig. 15M–O); 5.88 × 2.00 mm (RGM 1310233, Fig. 15P); 5.55 × 1.97 mm (RGM 1310236, Fig. 15Q).

########## Description.

Slender elongate shell, with up to nine convex whorls. Protoconch forms small bulbous cap, consisting of 1.3 whorls that measure 365 µm in diameter; surface weakly granulate, spiral striae set in after 0.5 whorls; nucleus is 140 µm wide; P/T boundary marked by thin, sharp axial line. Early teleoconch whorls have low convex profile. Two morphotypes are present: form A is broader, with whorls increasing slightly more in height (thus producing relatively larger last whorl) and little convex whorls; form B is more slender, whorls increase less fast in height in relation to width and whorl profile is stronger and more regularly convex. Both types are linked via intermediates. Generally, whorl profile varies between regularly convex (of varying strength), laterally flattened or bipartite (with near straight-sided upper half and convex lower half; rarely, transition between halves coincides with spiral thread). Suture is narrow. In some specimens, last whorl is slightly inflated and aperture is expanded. Traces of spiral sculpture, ranging from faint lines to blunt keels of variable number occur on several shells. Aperture expansion and sculpture are found on both morphotypes, as well as in intermediates. Umbilicus mostly covered by inner lip; if open, it is very narrow. Growth lines markedly sigmoidal, with prosocline upper third and opisthocline lower two-thirds.

########## Discussion.

The huge morphological variability with intergrading morphotypes complicates reasonable taxonomic distinctions within this taxon. Moreover, much of the shape variation (especially in later whorls) seems to be a result of shell repair after predator-induced damage.

The variability also hampers linking our material to an existing name. Several species (and varieties) have been introduced for slender elongate, multi-whorled shells from the Caspian Sea. While the sculptured representatives can be fairly well delimited, the smooth-shelled taxa have caused considerable confusion. Particularly challenging are the many small, slender species with pointy apex, moderately to strongly convex whorls and thin peristome. The group includes (aside from *T. spica*): *T. elegantula* (Clessin & W. Dybowski in W. Dybowski, 1887), *T. turricula* (B. Dybowski & Grochmalicki, 1915), *T. nossovi* (Kolesnikov, 1947), *T. concinna* (Logvinenko & Starobogatov, 1969), *T. spasskii* (Logvinenko & Starobogatov, 1969), *T. uralensis* (Logvinenko & Starobogatov, 1969) and *T. astrachanica* (Pirogov, 1971). *Turricaspia lyrata* (B. Dybowski & Grochmalicki, 1915), which was originally introduced as subspecies of *T. spica*, can be well delimited from that group because of its much larger, blunt apex.

A major problem in identifying and discriminating those species is that the concepts applied by later authors occasionally diverge largely from the original perceptions. This especially regards *T. spica* and the species described by B. Dybowski and Grochmalicki (1915). Unfortunately, the types for these species are not known for sure (Kantor and Sysoev 2006, Vinarski and Kantor 2016) and the original descriptions, drawings, and illustrations are mostly insufficient to allow distinction. Beyond that, different traits have been considered as diagnostic by different authors when describing new species, and morphological variability was hardly considered at all.

The identity of *Turricaspia spica* (sensu Eichwald) is dubious. The original description and illustration do not allow distinction from other similar species. The present specimens differ slightly from *T. spica* sensu Kantor & Sysoev, 2006, which is characterized by a faster whorl accretion rate and relatively higher whorls (including the last whorl). In contrast, our material largely fits the concept of *T. spica* as used by B. Dybowski and Grochmalicki (1917). We tentatively classify the Selitrennoye specimens in *Turricaspia spica*, being the oldest available name of the group. Many of the later proposed names might turn out to be junior synonyms. A more in-depth study is required to solve this problematic case.

########## Distribution.

*Turricaspia spica* is endemic to the Caspian Sea. After Logvinenko and Starobogatov (1969), it occurs at a water depth between 0 and 30 m, but those authors applied a different concept of the species.

######## Hydrobiidae incertae sedis

######### Genus Abeskunus

Taxon classificationAnimaliaLittorinimorphaHydrobiidae

Kolesnikov in Logvinenko & Starobogatov, 1969

########## Type species.

*Paludina exigua* Eichwald, 1838; by original designation. Caspian Sea, Pleistocene.

########## Discussion.

The genus *Abeskunus* and the species that have been attributed to it have caused considerable confusion. A detailed discussion of the taxonomic and nomenclatural problems associated with *Abeskunus*, considerations on its systematic placement, as well as a description of the type species will be provided in a forthcoming study. Preliminary work confirms classification of the species described below in *Abeskunus*.

######### Abeskunus brusinianus

Taxon classificationAnimaliaLittorinimorphaHydrobiidae

(Clessin & W. Dybowski in W. Dybowski, 1887)

Fig. 16A–I

*1887 Zagrabica Brusiniana nob.; W. Dybowski: 52–53.
1888 Zagrabica Brusiniana n. sp. – W. Dybowski: 79, pl. 2, fig. 7.
1952 Zagrabica brusiniana W. Dyb., 1888. – Zhadin: 235, fig. 166 [partim].
1969 Pseudamnicola [(Abeskunus)] brusiniana (Cless. et W. Dyb.). – Logvinenko & Starobogatov: 381, fig. 367 (15).
2006 Pseudamnicola brusiniana (Clessin et W. Dybowski in W. Dybowski, 1888). – Kantor & Sysoev: 114, pl. 51, fig. J.
2016 Pseudamnicola brusiniana (Clessin et W. Dybowski in W. Dybowski, 1888). – Vinarski & Kantor: 222.

########## Material.

489 specimens (RGM 1309834, RGM 1309842, RGM 1310194, LV 201505).

########## Type material.

Not traced.

########## Type locality.

“Kaspi-See” (Caspian Sea, no further details mentioned).

########## Dimensions.

**Figure 16.**
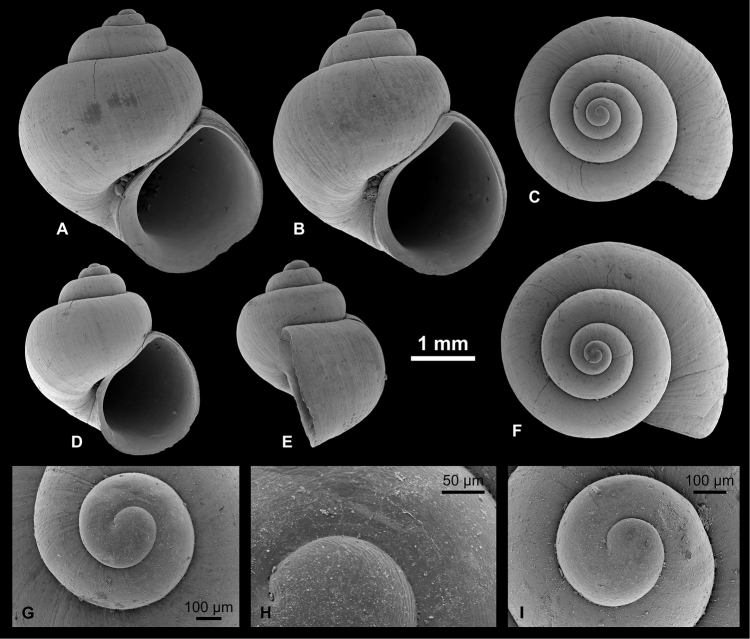
Hydrobiidae incertae sedis. **A, F** *Abeskunus brusinianus* (Clessin & W. Dybowski in W. Dybowski, 1887), RGM 1309834 **B, C, I** *A. brusinianus*, LV 201505 **D, E, G, H** *A. brusinianus*, RGM 1309842.

4.12 × 3.82 mm (RGM 1309834, Fig. 16A, F); 4.15 × 3.65 mm (LV 201505, Fig. 16B, C, I); 3.00 × 2.74 mm (RGM 1309842, Fig. 16D, E, G, H); 4.14 × 3.42 mm; 4.15 × 3.53 mm; 4.34 × 3.79 mm; 4.39 × 3.87 mm; 4.59 × 3.68 mm.

########## Description.

Shell broadly conical, comprising up to 4.5 whorls. Protoconch broadly domical, with almost immersed initial part; consists of 1.25 whorls, measures 525 µm in diameter; nucleus is ca. 160 µm wide; nucleus surface covered with irregular elongated wrinkles; protoconch surface wrinkled, bearing thin, irregular spiral grooves on first third, passing over irregular pattern of faint spiral grooves and wrinkles into numerous, regularly parallel spiral furrows on last third; P/T transition without growth rim, marked by onset of growth lines. Teleoconch whorls highly convex, with maximum convexity in adapical half, producing slightly stepped spire. Last whorl attains 77–85% of shell height. Aperture drop-shaped, slightly inclined, with marked adapical notch at contact to penultimate whorl. Outer peristome margin not or slightly thickened, columellar and parietal margins weakly thickened; peristome slightly expanded towards columella (protruding towards umbilicus in lateral view) and strongly towards base; weakly sinuate in lateral view, with broad but weak adapical protrusion and straight-sided abapical part. Umbilicus narrow, slit-like. Prosocline growth lines cover shell surface.

########## Discussion.

The species differs from the type species *A. exiguus* (Eichwald, 1838) in the conical shell, the taller spire, the less inflated last whorl, and the distinct umbilicus. *Abeskunus brusinianus michelae* Tadjalli-Pour, 1977 is much more globular than *A. brusinianus*. The latter species strongly reminds of and might be conspecific with *A. exiguus*. *Pseudamnicola depressispira* Logvinenko & Starobogatov, 1969, which these authors also included in the subgenus Abeskunus, differs from the presumed congeners in the valvatoid shape with very wide umbilicus and small but distinct riblets.

Pseudamnicola ? brusiniana Pavlović, 1903 is a junior secondary homonym of this species, for which Neubauer et al. (2015b) introduced Pseudamnicola ? babindolensis as replacement name. Because of the revised classification, P. ? brusinianus [sic] Pavlović is reinstated as valid, with P. ? babindolensis as its junior objective synonym (ICZN 1999, Art. 59.4).

########## Distribution.

Endemic to the Caspian Sea, in the southern and middle part at a depth of >250 m (Logvinenko and Starobogatov 1969, Parr et al. 2007).

### Non-indigenous species

In addition to the Pontocaspian elements, six taxa including ubiquitous Palearctic species were identified. They all occur in low numbers and differ from Pontocaspian species in their preservation state. Shells of larger taxa (*Valvata*, *Esperiana*, and *Lithoglyphus*) are eroded and suggest transport. The smaller planorbids are better preserved but differ in their orange color indicating that they were not deposited along with the whitish shells of the Pontocaspian residents. Moreover, all six taxa are typical freshwater dwellers (e.g., Welter-Schultes 2012). They probably derive from rivers flowing into the northern Caspian Sea (Fig. 1).

**Figure 17.**
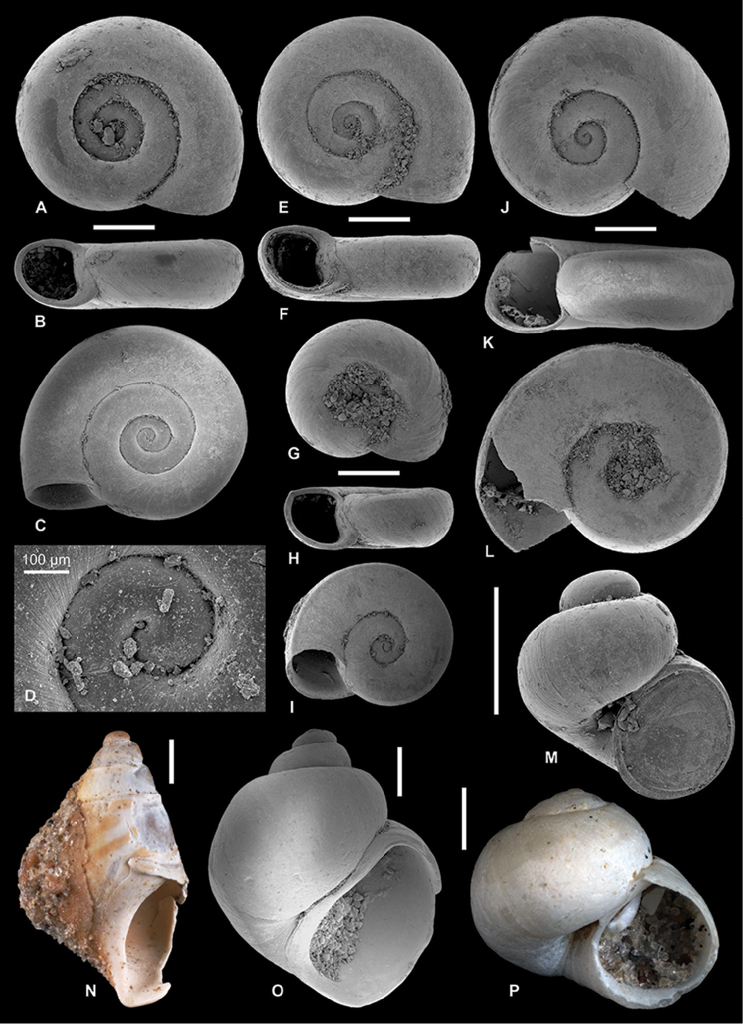
Non-indigenous species. **A–D** Anisus cf. spirorbis (Linnaeus, 1758), LV 201503 **E–F** A. cf. spirorbis, RGM 1309801 **G–I** Planorbis cf. planorbis (Linnaeus, 1758), LV 201504 **J–L** P. cf. planorbis, RGM 1309835 **M** *Bithynia* sp., juvenile, RGM 1309853 **N** *Esperiana esperi* (Férussac, 1823), RGM 1309792 **O** *Lithoglyphus naticoides* (Pfeiffer, 1828), RGM 1309832 **P** *Valvata piscinalis* (Müller, 1774), RGM 1310249. Scale bar equals 1 mm unless indicated otherwise. Note that all Planorbidae are figured at the same scale to facilitate comparison.

Anisus cf. spirorbis (Linnaeus, 1758) (Fig. 17A–F). – 13 juvenile to semi-adult specimens have been found. They match *A. spirorbis* as depicted by Glöer (2002) and Welter-Schultes (2012) in size, the regularly striated surface, the slightly overlapping whorls, and the weakly asymmetrical lateral profile. However, whorls expand in relative width a bit more rapidly in the Selitrennoye specimens, which is why we only tentatively assign our material to this species.

Planorbis cf. planorbis (Linnaeus, 1758) (Fig. 17G–L). – Two juvenile specimens and one semi-adult are available, showing either a distinct or intentions of a keel on the periphery of the apical side, a feature typical of *P. planorbis* (Glöer 2002, Welter-Schultes 2012). In addition, shell size, whorl expansion and lateral profile fit well to this species. Since no adult specimen with fully developed keel have been found, we attribute our specimens to this species provisionally.

*Bithynia* sp. (Fig. 17M). – Four juvenile specimens are available, consisting of the protoconch and about one teleoconch whorl; the operculum is in-situ preserved in all specimens. The classification is based on the presence and shape of the operculum, the characteristics of the protoconch, as well as the shape of the aperture, all of which are features typical of the genus *Bithynia* (compare Glöer et al. 2005, Neubauer et al. 2016b).

*Esperiana esperi* (Férussac, 1823) (Fig. 17N). – The single fragmentary specimen matches well the specimens illustrated by Welter-Schultes (2012). The similar and often co-occurring *Microcolpia daudebartii acicularis* (Férussac, 1823) is more elongate and lacks the color pattern (Glöer 2002, Welter-Schultes 2012). In the Russian literature, *E. esperi* is commonly listed as member of the genus *Fagotia* Bourguignat, 1884 (e.g., Starobogatov et al. 2004, Vinarski and Kantor 2016), which is, however, invalid as a junior objective synonym of *Esperiana*. Starobogatov et al. (1992, 2004) listed numerous species of *Fagotia* for extant European water bodies and categorized them into several subgenera. All of them are presently considered junior synonyms of *Esperiana* and *E. esperi*, respectively (for a complete synonymy list, see Vinarski and Kantor, 2016). *Fagotia roseni* Starobogatov in Starobogatov et al., 1992 from Quaternary deposits of Georgia also ranges within the variability of *E. esperi* and is herewith considered synonymous.

*Lithoglyphus naticoides* (Pfeiffer, 1828) (Fig. 17O). – The shape of the sole specimen ranges well within the large morphological variability of Recent *L. naticoides* (e.g., Glöer 2002). Late Pleistocene *Lithoglyphus jahni* Urbański, 1975 has a relatively taller conical shell and elevated spire (Kondrashov 2007). Coeval *Lithoglyphus pyramidatus* Möllendorf, 1873 is more elongate and lacks the stepped spire (Glöer 2002).

*Valvata piscinalis* (Müller, 1774) (Fig. 17P). – The eight, partly fragmented and corroded shells correspond well to Recent representatives of the species (Glöer 2002, Welter-Schultes 2012). Several of the *Valvata* species listed for the Volga delta region by Starobogatov et al. (1994, fig. 2) might be synonymous with this species. A conclusion on that matter requires examination of the material, which is unavailable to us.

## Discussion

The current work provides a first insight into the magnitude of endemic Caspian gastropod biodiversity. The gastropod fauna of Selitrennoye is composed of 24 species, 16 of which are Pontocaspian endemic species and 15 exclusively Caspian. Six species are considered to be non-indigenous based on the combination of a truly freshwater autecology, a general wide spread palearctic distribution and a slightly different preservation from the bulk of the well preserved Caspian lacustrine species in the material. The non-Caspian gastropods are low in numbers, and we suspect they may have either floated into the Caspian Sea during periods of high river discharge or, more likely, were mixed in from underlying sediment layers through bioturbation. The Selitrennoye fauna was deposited in open lacustrine settings at a paleosalinity of approximately 10–11 psu as suggested by the general composition of the mollusk fauna (Yanina 2012). The presence of paired bivalves in the same sample indicates the in-situ character of the fauna. The shelly levels are located around 17 m b.s.l., and late Khazarian maximum sea levels are estimated as 10 m b.s.l. The presence of very sandy sediments with lenses suggests deposition above storm wave base. Altogether, this might translate into a sea floor at about 7 m water depth. The settings can be best compared with the present-day southermost part of the northern Caspian Basin.

The taxonomy and systematics of Caspian gastropods is very much in need of an update. The abundant and well-preserved material presented here has given an indication about the generic placements of species and the magnitude of species richness. When compared to the latest inventory of Caspian gastropods by Vinarski and Kantor (2016), who presented 92 species for the entire Caspian Sea, our numbers (that represent a single locality) are still rather low. The synonymization of species we propose points in general to lower species numbers for Caspian gastropod faunas as reported before. However, the possibility exists that some of the species considered synonyms are sibling species. In order to test for that, we will require extensive new living material to perform combined genetic and morphometric analyses. In recent expeditions in the coastal areas of Azerbaijan and in the Caspian territory of Kazakhstan, we failed to detect living endemic Pyrgulinae gastropod species. All Caspian endemics are suffering badly from invasive species that have caused a total turnover of the fauna during the 20^th^ century (Kosarev and Yablonskaya 1995, Grigorovich et al. 2003, Orlova et al. 2005, Therriault et al. 2004, Riedel et al. 2006, Heiler et al. 2010, Albrecht et al. 2014). This situation appears to complicate or even make it impossible to follow such an integrated approach. Especially for the genera *Clessiniola*, *Laevicaspia*, and *Turricaspia*, we think devoted taxonomic revisions will be required to assess the number of species and potential presence of siblings.

The present revision does elucidate generic concepts. Even though it is open for further improvement, it will provide a basis for the establishment of evolutionary relationships within genera by comparison with older (Bakunian/Apsheronian) and younger Caspian faunas. By understanding species richness and evolutionary relationships of Caspian faunas, we will be able to document the nature and severity of the Anthropocene biodiversity crisis in this long-lived lake.

**Table 1:** List of species recovered from the late Khazarian deposits at Selitrennoye, with indication of their status as endemic to the Caspian Sea and the Pontocaspian region as a whole.

Species	Family	No. of specimens	Caspian endemic	Pontocaspian endemic
*Theodoxus pallasi* Lindholm, 1924	Neritidae	294		
*Ulskia ulskii* (W. Dybowski & Clessin in W. Dybowski, 1888)	Hydrobiidae	19	x	x
*Andrusovia brusinai* Starobogatov, 2000	Hydrobiidae	39	x	x
Ecrobia cf. grimmi (Clessin in W. Dybowski, 1888)	Hydrobiidae	345		
*Clessiniola variabilis* (Eichwald, 1838)	Hydrobiidae	4867		x
*Laevicaspia caspia* (Eichwald, 1838)	Hydrobiidae	300	x	x
*Laevicaspia cincta* (Abich, 1859)	Hydrobiidae	174	x	x
*Laevicaspia conus* (Eichwald, 1838)	Hydrobiidae	1135	x	x
*Laevicaspia kolesnikoviana* (Logvinenko & Starobogatov in Golikov & Starobogatov, 1966)	Hydrobiidae	514	x	x
*Laevicaspia vinarskii* sp. n.	Hydrobiidae	9	x	x
*Turricaspia andrussowi* (B. Dybowski & Grochmalicki, 1915)	Hydrobiidae	3	x	x
Turricaspia ? dimidiata (Eichwald, 1838)	Hydrobiidae	1	x	x
*Turricaspia lyrata* (B. Dybowski & Grochmalicki, 1915)	Hydrobiidae	562	x	x
*Turricaspia meneghiniana* (Issel, 1865)	Hydrobiidae	248	x	x
*Turricaspia pulla* (B. Dybowski & Grochmalicki, 1915)	Hydrobiidae	186	x	x
*Turricaspia pullula* (B. Dybowski & Grochmalicki, 1915)	Hydrobiidae	1	x	x
Turricaspia ? spica (Eichwald, 1855)	Hydrobiidae	1420	x	x
*Abeskunus brusinianus* (W. Dybowski & Clessin in W. Dybowski, 1888)	Hydrobiidae	489	x	x
*Valvata piscinalis* (Müller, 1774)	Valvatidae	8		
*Esperiana esperi* (Férussac, 1823)	Melanopsidae	1		
*Bithynia* sp.	Bithyniidae	4		
*Lithoglyphus naticoides* (Pfeiffer, 1828)	Lithoglyphidae	1		
Anisus cf. spirorbis (Linnaeus, 1758)	Planorbidae	13		
Planorbis cf. planorbis (Linnaeus, 1758)	Planorbidae	3		

## Author contributions

FPW and TY conducted field work and collected the material; SV and FPW processed the material; TAN, SV, and FPW identified the species; TAN and FPW wrote the manuscript.

## Supplementary Material

XML Treatment for Theodoxus

XML Treatment for Theodoxus pallasi

XML Treatment for Caspiinae

XML Treatment for Ulskia

XML Treatment for Ulskia ulskii

XML Treatment for Andrusovia

XML Treatment for Andrusovia brusinai

XML Treatment for Ecrobia

XML Treatment for Ecrobia cf. grimmi

XML Treatment for Pyrgulinae

XML Treatment for Clessiniola

XML Treatment for Clessiniola variabilis

XML Treatment for Laevicaspia

XML Treatment for Laevicaspia caspia

XML Treatment for Laevicaspia cincta

XML Treatment for Laevicaspia conus

XML Treatment for Laevicaspia kolesnikoviana

XML Treatment for Laevicaspia vinarskii

XML Treatment for Turricaspia

XML Treatment for Turricaspia andrussowi

XML Treatment for Turricaspia ? dimidiata

XML Treatment for Turricaspia lyrata

XML Treatment for Turricaspia meneghiniana

XML Treatment for Turricaspia pulla

XML Treatment for Turricaspia pullula

XML Treatment for Turricaspia ? spica

XML Treatment for Abeskunus

XML Treatment for Abeskunus brusinianus
